# Composite selection signals can localize the trait specific genomic regions in multi-breed populations of cattle and sheep

**DOI:** 10.1186/1471-2156-15-34

**Published:** 2014-03-17

**Authors:** Imtiaz Ahmed Sajid Randhawa, Mehar Singh Khatkar, Peter Campbell Thomson, Herman Willem Raadsma

**Affiliations:** 1 ReproGen - Animal Bioscience Group, Faculty of Veterinary Science, University of Sydney, 425 Werombi Road, Camden NSW 2570, Australia

**Keywords:** Selection signatures, Selective sweeps, Polledness, Double muscle, Geographic origin, Cattle, Sheep

## Abstract

**Background:**

Discerning the traits evolving under neutral conditions from those traits evolving rapidly because of various selection pressures is a great challenge. We propose a new method, composite selection signals (CSS), which unifies the multiple pieces of selection evidence from the rank distribution of its diverse constituent tests. The extreme CSS scores capture highly differentiated loci and underlying common variants hauling excess haplotype homozygosity in the samples of a target population.

**Results:**

The data on high-density genotypes were analyzed for evidence of an association with either polledness or double muscling in various cohorts of cattle and sheep. In cattle, extreme CSS scores were found in the candidate regions on autosome BTA-1 and BTA-2, flanking the *POLL* locus and *MSTN* gene, for polledness and double muscling, respectively. In sheep, the regions with extreme scores were localized on autosome OAR-2 harbouring the *MSTN* gene for double muscling and on OAR-10 harbouring the *RXFP2* gene for polledness. In comparison to the constituent tests, there was a partial agreement between the signals at the four candidate loci; however, they consistently identified additional genomic regions harbouring no known genes. Persuasively, our list of all the additional significant CSS regions contains genes that have been successfully implicated to secondary phenotypic diversity among several subpopulations in our data. For example, the method identified a strong selection signature for stature in cattle capturing selective sweeps harbouring *UQCC*-*GDF5* and *PLAG1*-*CHCHD7* gene regions on BTA-13 and BTA-14, respectively. Both gene pairs have been previously associated with height in humans, while *PLAG1*-*CHCHD7* has also been reported for stature in cattle. In the additional analysis, CSS identified significant regions harbouring multiple genes for various traits under selection in European cattle including polledness, adaptation, metabolism, growth rate, stature, immunity, reproduction traits and some other candidate genes for dairy and beef production.

**Conclusions:**

CSS successfully localized the candidate regions in validation datasets as well as identified previously known and novel regions for various traits experiencing selection pressure. Together, the results demonstrate the utility of CSS by its improved power, reduced false positives and high-resolution of selection signals as compared to individual constituent tests.

## Background

Genetics research has increased rapidly with availability of high throughput molecular biology tools and analytical approaches [1]. Recent molecular genetics techniques combined with large scale *in silico* analysis of genetic polymorphism data have provided insights to many questions about the origin of species [2], evolution [3], co-evolution and selection [4], domestication [5], genetic control of adaptation and diseases [6-8], and genetic diversity [9,10] for a wide range of species. More recently, identification of chromosomal regions that contain signatures of selection has been helpful to understand various mechanisms of adaptation, domestication and selection for important traits of various domestic species [11-21].

Evidence of selection can be gained from the measures of population differentiation, the allele frequency spectrum, linkage disequilibrium (LD) and haplotype structures [22,23]. Multiple methods have been developed for detecting selection signatures from genomic sequences and single nucleotide polymorphism (SNP) data [24,25]. Popular methods to capture selection evidence among populations from genetic polymorphism data include fixation index (*F*_ST_) [26,27], change in derived allele frequencies (ΔDAF) [23], allele frequency differences [28], long range haplotype (LRH) tests based on the extended haplotype homozygosity (EHH) statistic [29] including the across population extended haplotype homozygosity (XP-EHH) [22] and Rsb [30]. The specificity of each selection test statistic is limited to test certain aspects of selective forces operating under various models of natural and artificial selection. Hence, various selection tests being used often provide differing results for the same genomic dataset and likely none of these can exclusively provide a definite conclusion about the selective hypotheses [31].

Populations undergoing directional or divergent selection for specific traits are expected to exhibit signals of selection at the underlying genomic regions when measured by several selection tests [32]. Therefore, a combination of multiple strategies can be a robust approach in localizing such selected regions and correlating them with phenotypic variation. Several approaches to combine multiple summary statistics have been implemented that improve the power of detecting selection signatures [16,23,31,33,34]. Grossman et al. [23] developed a Bayesian estimator, composite of multiple signals (CMS), that combines several statistics to localize causal variants of positive selection. CMS requires extensive simulations and knowledge of the population genetic history to explore selection events under robust models with their underlying assumptions [15]. Success of CMS depends on the availability of very dense SNP data (for example, > 3 million SNPs in the human 1000 Genomes Project) required to approximate all the genome-wide functional variants. Lin et al. [31] and Pavlidis et al. [33] used machine learning methods implementing boosting and support vectors, respectively, which combines multiple statistics to maximize their joint predictive performance. They too require prior information from the estimates of population genetic diversity along with powerful computation platforms. Other efforts have also been made by combining selection signatures with association analysis in multiple species, however, these require information of phenotypes on individuals and in some cases also about their progeny [12,34,35]. Recently, Utsunomiya et al. [16] employed the Stouffer weighted Z-method [36] for combining *p*-values of several selection tests in their so called Meta-SS (meta-analysis of selection signals). Their assumptions to retrieve *p*-values directly from the test statistics require that each constituent test follow (approximately) a normal distribution, centred on zero under the null hypothesis if no selection. The implementation of Meta-SS is, therefore, limited to selected tests and incompatible on some popular selection tests such as *F*_*ST*_ where the distribution (under the null hypothesis) is not known. The limitations and complexity of methods, prior information, high-density genotypes and powerful computational resources required to implement available combining approaches leaves researchers with limited resources at a disadvantage.

Understanding the genetic control of heritable phenotypes is decisive to implement strategies for the rapid improvement in the qualitative and quantitative features of any domesticated species. Owing to the high genetic diversity in cattle and sheep, with over 800 and 1400 breeds, respectively, and substantive known factors for shaping their genetic diversity, they have been extensively used as model species for exploring selection signatures [11-21,32,37-42].

In general, genetically alike populations are expected to share genetic polymorphism at the genomic regions carrying genes for common phenotypes, whereas, genetically isolated populations may have uniquely positioned or divergent patterns of polymorphism on the genome [11,15,43]. Combining genotypic data on multi-population panels for identical traits has been used successfully to estimate the genomic breeding values and genomic selection [44,45], local adaptation [43], phylogeography and breeding history [11,46], and association mapping [47]. Therefore, detection of signatures of strong selection can be boosted by combining samples from multiple breeds based on known traits and compare such multi-breed populations for the contrasting phenotypes [12,15,48]. Across phenotypic groups, the contrast in genetic variation at the putative genomic regions increases the likelihood of capturing the selection signatures linked to the traits of interest. Within groups, the genome-wide genetic diversity between multiple breeds will lower background noise (false positive signals) which have accumulated confounding genetic patterns due to the demographic history of breeds or by the random genetic drift [47].

In principle, a simple method to combine outputs from separate tests based on their statistical distributions can be used to increase the accuracy of linking genotypes (genomic regions) with phenotypes without prior information on population history, individual phenotypes or genetic relationships. Here we present an improvement in the trait-specific genome-wide scans based on SNP data to map selection signatures by unifying multiple information from: i) evidence of selection, and ii) phenotypically alike populations. We developed a composite index of selection signatures: composite selection signals (CSS), and tested this against phenotypes controlled by known major genes in cattle and sheep. In addition, we investigated European and African *Bos taurus* cattle to identify the signatures of selection in geographically isolated populations.

## Methods

### DNA samples and genetic polymorphism data

Utility of the composite selection signal was tested in cattle and sheep by analyzing data available from various published studies on both species. To add power by increasing the sample size and to maximize the range of breeds and animals within breeds, samples collected by independent research groups were merged. Cattle data consisted of 1,096 animals representing 56 cattle breeds as described in previous studies [3,10,39,49]. Genetic relationships from the genome-wide SNPs were estimated by computing a genome-wide IBS matrix using PLINK [50] to identify and remove duplicate samples across multiple datasets of cattle. The sheep dataset consisted of 2,803 animals from 74 breeds [11]. The samples and breeds of cattle and sheep included in this study are listed in (Additional file 1: Table S1) and (Additional file 2: Table S2), respectively.

SNP genotypes generated in previous studies on cattle [3,10,39,49] and sheep [11] genotyped with the Illumina BovineSNP50 chip and Illumina OvineSNP50 chip assays, respectively, were used in the present analysis. After quality control, 38,610 and 47,502 autosomal SNPs were retained for cattle and sheep, respectively (Additional file 3: Table S3), and the final number of heterozygous SNPs (minor allele frequency (MAF) > 0.01) in each dataset is given in Table 1. Imputation of sporadic missing genotypes and haplotype phasing was performed with BEAGLE 3.3 [51]. Ancestral alleles were inferred for cattle genotype data using information from Matukumalli et al. [52] and, when possible, using information from the genotypes of three out-group species (bison, buffalo and yak) from Decker et al. [3]. All SNPs were mapped on the UMD3.1 bovine genome assembly (http://www.cbcb.umd.edu/research/bos_taurus_assembly) and OARv1.0 ovine genome assembly (http://www.livestockgenomics.csiro.au/sheep/oar1.0.php) for the corresponding species.

**Table 1:** Breeds, samples, genotypes (SNPs) and known genes in each group of cattle and sheep

**Species**	**Trait**	**Groups**	**Breeds (*n*)**^**a**^	**Animals (*n*)**	**Genome assembly**	**SNPs (*n*)**^**a**^	**SNP density (kb)**	**Derived SNPs (*n*)**	**Known genes**	**Dataset code**
**Cattle**	Polledness	Poll head	7	85	UMD3.1	38,290	65.50	38,177	*POLL* locus	**A**
		Horn head	7	127						
	Double muscling	Double muscling	3	49	UMD3.1	38,520	65.15	38,407	*MSTN*	**B**
		Normal muscling	14	308						
**Sheep**	Polledness	Poll head	37	1489	OARv1.0	47,498	51.26	-	*RXFP2*	**C**
		Horn head	36	1290						
	Double muscling	Double muscling	3	149	OARv1.0	47,502	51.26	-	*MSTN*	**D**
		Normal muscling	71	2654						
**Cattle**	Geographic location	African	7	226	UMD3.1	37,905	65.67	37,795	-	**E**
		European	46	847						

^a^Details of breeds and genotyping information about cattle and sheep is available in the (Additional files 1, 2 and 3: Table S1, S2 and S3, respectively).

### Phenotype data

Two subsets from both the cattle and the sheep data, collectively called as validation datasets (A-D), were extracted based on traits known to be under control of a major autosomal gene, namely double muscling (increased skeletal muscle mass) and polled (absence of horns) phenotypes (Table 1). In cattle, the dataset A consisted of animals of seven polled breeds and seven horned breeds. The dataset B of cattle consisted of animals from three double muscle breeds and 14 normal muscle beef breeds. In sheep, the dataset C contained animals from 37 naturally polled sheep breeds and 36 horned sheep breeds and the dataset D had data on animals from three breeds known to be double muscled and 71 breeds without the double muscle phenotype.

Candidate genes for the two traits in validation datasets (A-D) of both species are described as follows:

#### Polledness in cattle

*POLL* locus is located at the proximal end of bovine autosome 1 (BTA-1) at 1.65-2.05 Mb position. The dominant alleles of causal mutations in the genes harbouring the *POLL* locus cause the polledness in cattle [15,17,20,47,52,53].

#### Double muscle in cattle

Bovine *Myostatin* (*MSTN*) i.e. *growth and differentiation factor 8* (*GDF8*) gene (BTA-2: 6213566 – 6220196 bp) harbours various alike-in-state mutations in its third exon that underlie the muscular hypertrophy (a partially recessive trait) in some beef cattle breeds. For example, the double muscles are linked to the loss-of-function substitution in Piedmontese (and rarely in other beef breeds) and a frame-shifting 11 nucleotide deletion in Belgian Blue, South Devon and Asturiana de los Valles [20,39,54,55].

#### Polledness in sheep

*Relaxin/insulin-like family peptide receptor 2* (*RXFP2*) gene on ovine autosome 10 (OAR-10: 29491481 – 29538132 bp) is located in a known selected genomic region linked to the horn morphology in sheep [11,56,57].

#### Double muscle in sheep

Ovine *MSTN* gene on OAR-2 (126318371 – 126323354 bp) harbours a single loss-of-function mutation in its 3′-untranslated region (strongly selected in Texel) that inhibits its translation resulting the double muscle in sheep [11,55,58].

In addition, for dataset E, cattle breeds of European (46 breeds, 847 animals) and African (7 breeds, 226 animals) origin were compared (Table 1). There were several cattle breeds of small sample size (*n* < 20) in the European group. Therefore, the effect of sample size on the computation of our composite and constituent selection tests was also assessed by comparing results from analyses by excluding and including the breeds with small sample size (*n* < 10 and *n* < 20).

### Test statistics for selection signatures

The signatures of recent positive selection are expressed as a localized increase in allelic frequency of the beneficial mutations towards fixation in the population. Non-ancestral alleles at mutated loci are called “derived” alleles and usually, the function-altering derived alleles create the phenotypic diversity. The excess of recently selected beneficial (ancestral or derived) alleles results in a ‘hitchhiking’ of neighbouring polymorphisms which results in extended haplotype homozygosity in the region of selection [22]. We selected three single test statistics which capture the increase in highly differentiated loci (*F*_ST_), or increase in derived allele frequency (ΔDAF and ΔSAF), or the increase in haplotype homozygosity (XP-EHH) along the genome in each of the five datasets. A brief implementation of each test statistic is described below. The new method, which we term as composite selection signal (CSS), combines the three estimates of the single selection tests in a single index.

#### *F*_ST_

The fixation index (*F*_ST_) of population differentiation is estimated from the deviation in allele frequency between populations compared against the within population polymorphic frequency [26]. It can detect selection signatures using genetic polymorphism data by a pairwise comparison between two contemporary populations. SNP-specific *F*_ST_ values were computed for each pair of phenotypically contrasting groups within all the sets of cattle and sheep data using a custom R script available upon request. Extreme positive values of *F*_ST_ for the particular locus are indicative of high levels of reproductive isolation of the two populations and divergent selection in both or strong positive selection in one of the populations and/or random drift.

#### ∆ ***DAF***

Highly differentiated SNPs with an excess of new mutations (derived alleles) can be identified by the distribution of derived allele frequency (DAF). Change in the DAF (∆DAF) was calculated as the difference of DAF in the putative selected population or group (D_S_) and the DAF in the alternative non-selected populations or groups (D_NS_), where ΔDAF = D_S_ − D_NS_ as given in Grossman et al. [23]. ∆DAF scores have an approximate normal distribution. We standardized ∆DAF to have a zero mean and unit variance to identify the outlier SNPs. The use of the ∆DAF statistic was restricted to cattle data where the derived and ancestral allele could be inferred unambiguously. In sheep, no such out-group was available; hence, the ancestral allele could not be inferred.

#### ∆ ***SAF***

To accommodate the lack of information on ancestral allele in sheep, we developed a simple statistic based on the allele frequency differences between the populations. Based on the observed allele frequency distributions, we calculated the directional change in the selected allele frequency (∆SAF) across two populations *i* and *j*, so that ∆SAF $=f_{A_{i}}-f_{A_{j}}$, where, $f_{A_{i}}$ is the frequency of allele A, the major allele in the putatively selected population *i*; similarly, $f_{A_{j}}$is the frequency of allele A in non-selected population *j*. ∆SAF scores were also standardized to *Z* ~ *N*(0,1)*.* Since the estimates of ∆DAF and ∆SAF are a function of the allele frequency distributions, a significant association is expected for loci under strong selection and can be used alternatively depending on the availability of required information about derived and ancestral alleles. Comparison between ∆DAF and ∆SAF to validate the latter using the cattle data has shown a very strong correlation (*r* > 0.8) for the SNP scores at candidate gene regions and genome-wide. Replacement of ∆DAF by ∆SAF as input in CSS has shown no appreciable difference in the results for the control regions of cattle (data not shown).

#### XP-EHH

A multi-allelic (haplotype based) test has many advantages in studying genome-wide patterns of divergence over single locus (SNP) analyses, since the latter may be less informative due to ascertainment bias in the SNP discovery process [59]. Long-range haplotype (LRH) tests can detect the signals of positive selection by finding common alleles carried on unusually long haplotypes. Due to LD, selection pressure on a beneficial allele at a polymorphic locus can also affect the neighbouring neutral loci, resulting in long haplotypes of low diversity across extended regions [60]. Extended haplotype homozygosity (EHH) detects selection signatures by comparing a base (core) haplotype, characterized by high frequency and extended homozygosity, with other haplotypes at the selected locus. EHH is the probability that two randomly selected chromosomes carrying the candidate core haplotype are homozygous for the entire interval spanning the target region for a given locus. The EHH statistic depends on the allele frequency and the strength of LD with neighbouring loci; hence, it is applicable to an incomplete selective sweep when the selected allele becomes very frequent but is not yet fixed within a given population. EHH is less robust in a situation where the selected alleles may have reached fixation and their alternative alleles have disappeared in a population i.e., a complete selective sweep [43]. Complete selective sweeps can be dealt with using the across population EHH (XP-EHH) test, which compares each population (breed) with the other population(s) on corresponding haplotypes. XP-EHH has high power to detect selection signatures in small sample sizes and power may be gained by the grouping of genetically similar breeds [22,23,29,43]. We calculated the XP-EHH for each of the five datasets using the procedure described by Sabeti et al. [22]. Further, XP-EHH scores were standardized in each analysis so that a genome-wide distribution of all scores has zero mean and unit variance.

### Composite Selection Signals (CSS)

Three selection tests (*F*_ST_, XP-EHH, ΔDAF or ΔSAF) were combined with the hypothesis that a common signal across the multiple test statistics would be detected as an extreme CSS score at the trait specific genomic positions. The following outlines the method used to compute CSS scores from combining the three component test statistics for the same SNP, as well as determining *p*-values for these composite tests, to test for the existence of a common signal.

Let *T*_*ij*_ be the test statistic using method *i*, (*i* = 1, …, *m*) calculated at SNP *j*, (*j* = 1, …, *n*). Then for each test statistic type *i*, obtain the rank of each observed test statistic across all *n* SNPs, say *R*_*ij*_ = rank(*T*_*ij*_), which takes values 1, …, *n*. Next, these ranks are converted to fractional ranks by re-scaling them to lie between 0 and 1, i.e. *R*′_*ij*_ = *R*_*ij*_/(*n* + 1), giving values from 1/(*n* + 1) through *n*/(*n* + 1).

Note that the fractional rank does not use the magnitudes of the actual test statistics: this makes it inherently robust, as in any other nonparametric procedures that are based on ranks. However, there is therefore some loss of information. Some of this information may be recovered by converting the fractional ranks to *z*-statistics, *Z*_*ij*_ = Φ^− 1^(*R* '_*ij*_), where Φ^− 1^(·) is the inverse cumulative distribution function (CDF) for a standard normal, i.e. maps values 0 through 1 to an underlying standard normal distribution, *Z* ~ *N*(0,1). Once converted to normal scores, the average *z*-values were calculated at each SNP position, ${\bar{Z}}_{j}$, *j* = 1, …, *n*, and *p*-values were directly obtained from the distribution of means from a normal distribution, $\bar{Z}\sim N\left(0,m^{-1}\right)$, i.e. $p=1-\Phi \left(m^{1/2}{\bar{Z}}_{j}\right)$ where Φ(⋅) is the CDF for a standard normal distribution.

The log-transformed *p*-values (−log_10_*p*) corresponding to the set of mean *Z* values (${\bar{Z}}_{j}$) were declared as the composite selection signals (CSS) and these were plotted against the genomic positions to identify the significant selection signals. If there is a common signal across the multiple test statistics, this will show up as an excess in CSS at that point, otherwise, CSS may be dampened down, i.e., regressed to the genome-wide average.

### Significant SNPs under selection

The results from five datasets (Table 1) were compared across three constituent tests and CSS. In the absence of a known probability distribution for most cases of the test statistics used in this study, SNPs with extreme test scores (top 0.1%) in the genome-wide distribution were considered significant [11]. Selected variants tend to impose the selection pressure on neighbouring alleles because of hitchhiking; therefore, significant signals are expected to cluster together. Hence, in order to minimize the spurious noise from single SNP tests with resultant false positives, the test statistics were averaged (smoothed) over SNPs within 1 Mb sliding windows centred at each SNP along the chromosomes.

### Genomic regions and genes under selection

Clusters consisting of a multiple SNPs with the extreme CSS test statistics (top 0.1%) spanning 1 Mb windows around the SNP with most extreme value were selected. This was termed as a significant cluster by each test and its boundaries were defined by the first and last SNP. Consecutive clusters spaced less than 1 Mb apart were merged into a single cluster. Further, for mining candidate genes, we define the genomic regions underlying the significant clusters by including an additional 0.5 Mb on each side, considering genome-wide uniform LD patterns.

For comparison across multiple tests, we identify the genomic region by each test and count the numbers of significant SNP scores in other selection tests within each region. For example, at the first step, regions were defined by CSS and significant SNPs were counted in XP-EHH, *F*_ST_ and ΔDAF (or ΔSAF).

The significant genomic regions were investigated for genes that mapped on the respective genome assembly of both species for the candidate traits. For the genes in non-candidate regions identified by CSS, we further investigated the respective subpopulation for any additional phenotypes that might have been under positive selection. Similarly, genes underlying the significant genomic regions in geographic population groups of cattle were also investigated to understand the historic and commercial imprints of selection.

### False discovery rate

The control of false positive signals in multiple hypotheses testing is essential in genomic studies. The false discovery rate (FDR) is considered a reliable statistical method for correction in case of multiple comparisons. The estimation of FDR is influenced by the accuracy of the *p*-value estimations and the validity of their underlying distributional assumptions. Correctly estimated *p*-values from the null hypothesis are assumed to exhibit a uniform distribution. Usually, on the other hand, observed distribution of *p*-values from multiple tests consists of a mixture of distributions of *p*-values from true null hypotheses along with true alternative hypotheses. To improve the accuracy of FDR estimation, empirical *p*-values from non-smooth CSS were calibrated using the constrained regression recalibration (ConReg-R) method so that the observed *p*-values have the properties of an ideal empirical *p*-value distribution [61]. The tail area based FDR (*q*-values) were estimated from the calibrated *p*-values using the R package “fdrtool” [62] with its default options for “statistic = *p-value*”, when it uses the empirical data below the 75th percentile to determine the null distribution of the test statistics.

FDR were computed against the calibrated *p*-values for the raw CSS scores of each validation dataset analysis. Within the significant region boundaries, the percentages of SNPs having FDR ≤ 5% were calculated. To differentiate the distribution of true null and true alternate hypotheses, we compared the density distribution of FDR (*q*-values) of SNPs within significant regions against the rest of genome-wide SNPs.

## Results

### Identification of significant loci

**Figure 1:**
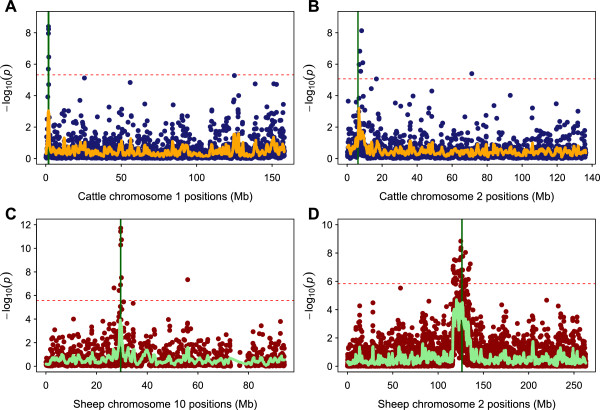
**Composite selection signals (CSS) for validation datasets.** Chromosome-wise plots of highest CSS scores are shown for trait-wise datasets of cattle **(A and B)** and sheep **(C and D)**. The dotted red horizontal lines in the CSS plots indicate the genome-wide 0.1% thresholds of the empirical scores. Smooth lines are the smoothed CSS scores by averaging SNPs within each 1 Mb window. Vertical green lines indicate the location of candidate genes at each chromosome as follows: **A** = *POLL locus* for polledness in cattle (dataset **A**), **B** = *MSTN* for double muscle in cattle (dataset **B**), **C** = *RXFP2* for polledness in sheep (dataset **C**), and **D** = *MSTN* for double muscle in sheep (dataset **D**).

The map of chromosomes containing highest empirical CSS scores within each trait-wise dataset (A to D) is presented in Figure 1. Genome-wide comparisons of empirical distributions of all the selection tests across the four validation datasets are shown in (Additional file 4: Figure S1), (Additional file 5: Figure S2), (Additional file 6: Figures S3) and (Additional file 7: Figure S4). A strategy of smoothing SNP-wise empirical statistics was applied to three component selection tests and composite selection signals: for each case, the mean number of SNPs in genome-wide 1 Mb windows was 17 and 19 SNPs in cattle and sheep data, respectively (Additional file 8: Figure S5). The windows containing fewer than 5 SNPs were discarded from further analysis. After pruning such low SNP density windows, 38,211 (dataset A) and 38,441 (dataset B) sliding windows were retained for polled and double muscle cattle, respectively. Similarly, 47,438 (dataset C) and 47,442 (dataset D) sliding windows of averaged (smooth) test statistics were used from the polled and double muscle sheep analyses, respectively.

Genome-wide low to moderate correlations among the pairs of three single tests suggest a partial concordance among these tests; whereas, CSS has a high correlation with its all component tests, which suggests capture of information across multiple tests (Additional file 9: Figure S6). The genome-wide map of empirical scores (non-smoothed) and smoothed scores indicates a number of genomic regions with clusters of SNPs with high scores in each of the four analyses.

The magnitude of smoothed CSS in the significant clusters was affected by the SNP density and extent of LD between the SNPs within the sliding window. For example, the *POLL* locus is located on the proximal end (rich crossing over region) of BTA-1 where the high recombination rate reduces the LD among neighbouring SNPs (Table 1). In dataset A, highly significant raw CSS scores were located in the candidate gene region on BTA-1 (Figure 1-A), whereas existence of strong LD (see Discussion) on BTA-14 has lifted this region to the top of the smoothed distribution as shown in the genome-wide distribution in (Additional file 4: Figure S1-A). In datasets B, C and D, in contrast to dataset A, the magnitude of raw as well as smoothed CSS scores remained on top in the genome-wide distribution because their candidate regions were localized in cold-spots of less frequent recombination (Additional files 5, 6, 7: Figure S2 to S4).

### Significant genomic regions under selection in validation datasets

Of the genome-wide smoothed test statistics, the top 39 and 48 SNPs (i.e. top 0.1%) in the cattle and sheep datasets, respectively were used to find significant regions under selection. A number of selection signals were found in each dataset by all the test statistics. Overall, 9, 12, 10 and 5 genomic regions were detected in datasets A, B, C and D, respectively (Additional file 10: Table S4). These multiple significant regions were the result of low concordance between the component tests and their power to capture slightly different characteristics of the selective sweep. Note that across the four datasets, 15, 15 and 21 genomic clusters were captured by XP-EHH, *F*_ST_ and ΔDAF/ΔSAF, out of which 4, 5 and 13 regions were specific to individual tests. These 36 regions were narrowed down to 12 significant regions with the CSS approach (Table 2).

**Table 2:** Genomic regions under selection in cattle and sheep identified using composite selection signals (CSS)

**Region**^**a**^	**Chr**	**Position**^**b **^**(Mb)**	**Number of significant SNPs**				**Total genes**^**c**^	**Known genes**^**d**^	**Gene function**
			***CSS***	***XPEHH***	***F***_***ST***_	***ΔDAF***			
**A1**	**1**	**1.01-2.63**	10*	**9**	**1**	**-**	**15**	***POLL locus***	**Polledness**
A5	13	63.90-65.97	18	23*	1	5	26	*UQCC, GDF5*	Stature
A7	14	23.78-25.61	11	7	5*	10*	12	*PLAG1, CHCHD7*	Stature
**B1**	**2**	**6.15-7.82**	10*	11*	**3**	**-**	**9**	***MSTN***	**Double muscle**
B2	6	66.55-68.11	11	8	-	-	6	*COX7B2, FRYL*	Reproduction
B6	16	44.49-46.05	11	11	1	-	12	*NMNAT1, PIK3CD, SPSB1, SLC*	Embryonic growth, immunity
B8	18	13.34-15.03	5	3	1	-	33	*MC1R*	Coat colour
**C5**	**10**	**28.54-30.05**	26*	17*	34*	5*	**9**	***RXFP2***	**Polledness**
C8	13	66.97-68.50	7	-	7	3	17	*ASIP*	Coat colour
C10	25	6.67-8.29	14	10	-	2	16	*LRP4*	Bone growth
D2	2	119.62-122.30	20	11*	10	16	26	-	-
**D4**	**2**	**124.25-128.05**	28*	**22**	**27**	**27**	**47**	***MSTN***	**Double muscle**

Cluster of a minimum of three significant SNPs within a window spanning 1 Mb genomic locations centred on a core SNP above the threshold (top 0.1%) in CSS (smoothed statistics) are reported and are compared with the constituent tests.

^a^Prefix (A, B, C and D) with each region number represents the dataset as defined in Table 1 and rows in **bold** indicate the genomic regions containing candidate genes. A complete list of 36 genomic regions, their positions, range of all significant clusters (for each test) and genes under clusters of significant SNPs is shown in [Additional file 10: Table S4].

^b^Position of genomic regions includes a 0.5 Mb extension on both sides of boundaries of the main cluster identified by CSS to compare constituent tests and count of genes (see Methods). Large sized (> 1 Mb) regions are formed by joining successive (<1 Mb apart) clusters.

^c^Genes mapped on bovine (UMD3.1) and ovine (OARv1.0) assemblies within the boundaries of genomic regions.

^d^Candidate genes with known functional/structural effects for a particular trait present in the contrasting panels of multiple breeds.

*Indicates the cluster of highest ranked SNPs (raw scores) for a particular selection test.

Regions identified through CSS were further investigated to find specific genes associated with positive selection. A number of genes were found in each region; therefore, precise inferences about the specific target of selection may be difficult. The results from the component tests suggest a high concordance for significant clusters in the candidate regions but also a number of additional significant signatures located in genomic regions of unrelated or unknown genes (Additional file 10: Table S4). The concordance between the three distinct tests statistics at the four control regions establishes the support of CSS for detecting true selection signatures. The CSS test has fewer significant clusters and most of these are close (where SNPs are missing within genes) or harbouring the genes associated with the traits of interest in all datasets. We briefly describe the genomic regions under selection identified from each dataset by CSS as follows:

### Signatures of selection in validation datasets

The genome-wide map of empirical scores (non-smoothed) indicates that the highest CSS above the 0.1% threshold were in the candidate regions in all of the four analyses (Figure 1). At least five significant SNPs for CSS were found for each trait within the respective genic regions. The three component tests (*F*_ST_, ΔDAF or ΔSAF, and XP-EHH) were found coinciding in the candidate gene regions but with fewer and lower ranked SNPs as compared to the CSS test.

In dataset A, significant CSS scores were found in the candidate region (BTA-1) harbouring the *POLL* locus for polledness in cattle (Figure 1-A, and region A1 in Table 2). Two additional significant clusters were found on BTA-13 (region A5: *UQCC-GDF5* genes) and BTA-14 (region A7: *PLAG1-CHCHD7* genes) (Table 2, Additional file 4: Figure S1-A).

In dataset B, the highest CSS scores were localized at BTA-2 flanking *MSTN*, the gene responsible for double muscling in cattle (Figure 1-B, and region B1 in Table 2). Additional peaks of significant CSS were located on BTA-6 (region B2: *COX7B2* gene and near *FRYL, PDGFRA* genes), BTA-16 (region B6: *SLC25A33* and *SLCC45A1* genes) and BTA-18 (region B8: *MC1R* gene) (Table 2, Additional file 5: Figure S2-A).

In dataset C, the candidate region on OAR-10 harbouring the *RXFP2* gene for polledness in sheep contained the extreme CSS scores (Figure 1-C, and region C5 in Table 2). In addition, OAR-13 (region C8: *ASIP* gene) and OAR-25 (region C10: *LRP4* gene) exhibit the one significant peak each (Table 2, Additional file 6: Figure S3-A).

In dataset D, extreme CSS scores were found flanking the *MSTN* gene for double muscling in sheep on OAR-2 (Figure 1-D, and region D4 in Table 2). Notably, both significant peaks are on OAR-2 and are in the candidate region or in LD with the candidate gene region spanning an 18 Mb region (Table 2, Additional file 7: Figure S4-A).

The non-candidate regions in datasets A, B and C, contain genes that have been previously linked to various phenotypes in several species. Some of these genes were associated with phenotypes within our subpopulations (see Discussion). Overall, presence of the significant clusters of extreme CSS scores in the candidate regions of the cattle and sheep cohorts indicates improved power of CSS as compared to the constituent individual tests.

### False discovery rate (FDR)

**Figure 2:**
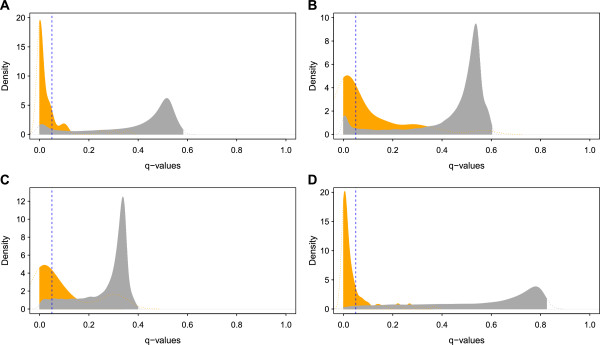
**Density distribution of false discovery rate (*q*-values) of SNPs in significant clusters (orange) and the rest of the genome-wide SNPs (gray).** Density plots are shown for polled cattle **(A)**, double muscle cattle **(B)**, polled sheep **(C)** and double muscle sheep **(D)**. Vertical dashed (−−−−−) lines indicate *q*-values (FDR) = 0.05 in each subset. *q*-values were calculated from the calibrated *p*-values. Histograms of the mean *Z*, empirical and calibrated *p*-values are shown in Additional file 7: Figure S4. Relationship between *q*-values and calibrated *p*-values is shown in Additional file 8: Figure S5.

While the distribution of *p*-values for regions without evidence of selection is not uniform (Additional file 11: Figure S7), there is nonetheless a clear ‘spike’ in the frequency of very small *p*-values, lending support for evidence of selection signatures. Nonetheless, genome-wide *q*-values were calculated for the calibrated *p*-values to estimate the FDR for each analysis (Additional file 11: Figure S7 and Additional file 12: Figure S8). Overall, the top 0.1% of SNPs based on raw CSS scores of the four datasets has considerably low FDR (*q* < 0.0001). Figure 2 shows a clear distinction between the density distributions of *q*-values for the SNPs in identified regions and SNPs in the rest of the genome for each dataset. Table 3 further shows that the identified genomic regions have a much higher proportion of SNPs with low *q*-values suggesting strong evidence for selection signals in the data. These proportions in the control regions in cattle are 85.7% (regions A1) and 90% (region B1) as compared to genome-wide proportions of 9.8% (dataset A) and 6.2% (dataset B), respectively. In sheep, 46.2% and 75.9% of total SNPs have *q* ≤ 0.05 in candidate regions C5 and D4 as compared to much lower values of 5.3% and 2.4% for datasets C and D, respectively for their neutral regions. Similarly, in all the non-candidate regions in the four datasets, the percentage of SNPs with *q* ≤ 0.05 is significantly higher as compared to the rest of the genome (Table 3).

**Table 3:** False discovery rates within identified genomic regions in each validation dataset of cattle and sheep

**Region in Table **2	**Chromosome**	**Total SNPs**	**SNPs in region *q*** **≤ 0.05**	**SNPs outside regions *q*** **≤ 0.05**
		***n***^**a**^	**%**	**% (in dataset)**
A1	1	14	85.7	9.8 (A)
A5	13	19	78.9	
A7	14	11	81.8	
B1	2	10	90.0	6.2 (B)
B2	6	11	63.6	
B6	16	11	36.4	
B8	18	12	41.7	
C5	10	26	46.2	5.3 (C)
C8	13	9	44.4	
C10	25	15	60.0	
D2	2	23	87.0	2.4 (D)
D4	2	54	75.9	

^a^Total number of SNPs located within the boundaries of the main cluster identified by CSS and their position exclude 0.5 Mb additions for gene investigation (shown in Table 2).

### Signatures of selection in geographically isolated multi-breed populations of cattle

**Figure 3:**
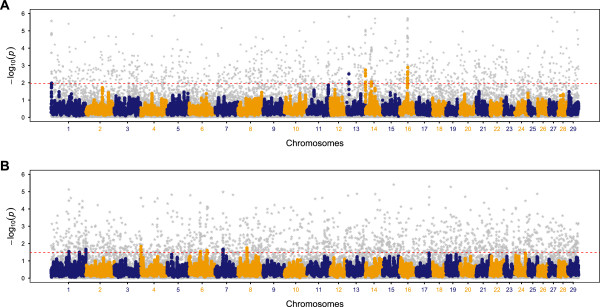
**Composite selection signals (CSS) for geographically isolated cattle populations.** Manhattan plots of -log_10_(*p*) of CSS are shown for **(A)** European *Bos taurus* and **(B)** African *Bos taurus.* Genome-wide smoothed CSS scores for SNPs on consecutive chromosomes are shown in various colours. Dotted red line in the CSS plots indicate the genome-wide 0.1% (upper cutoff) thresholds of the empirical smoothed scores. Gray stars are shown for raw CSS scores in the genome-wide background and bold at the putative selection regions underlying the significant clusters.

Finally, in dataset E, the smoothed scores from 37,827 sliding windows (after removing windows containing < 5 SNPs) were plotted along the genome in order to investigate the regions under selection. Figure 3 shows the Manhattan plots of smoothed CSS scores for European and African groups of *Bos taurus* cattle; complete list of significant genomic regions and underlying genes in both groups are listed in (Additional file 13: Table S5). The comparison of each test by including and excluding breeds with less than 10 and 20 animals showed negligible differences for the effect of variable sample size (especially low) of breeds in European group (Additional file 14: Figure S9). It shows that breeds with a similar history generally have shared patterns of genetic diversity. In addition, it also provides evidence that computation of CSS is not sensitive to the individual sample size of the participating breeds for outbred populations.

**Figure 4:**
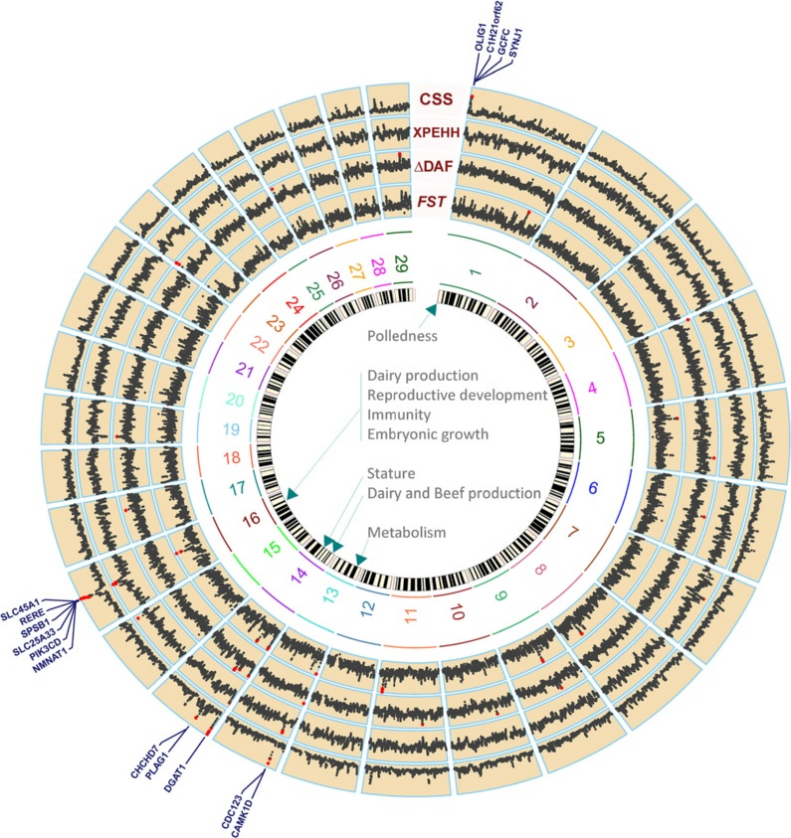
**Circos plot of genome-wide composite (CSS) and constituent (XPEHH, ΔDAF and *F***_***ST***_**) smoothed test statistics in European Bos *taurus *cattle.** Significant selection signatures in each test are highlighted with the red dots. Genes of important functions underlying the significant genomic regions identified by CSS are annotated and complete list of genes is available in (Additional file 13: Table S5). Circos plot was created using modified functions from the R package “RCircos” [63].

We note that, overall, CSS method identified clear peaks of higher magnitudes in European group as compared to the African cattle (Figure 3). The differences in the historical and recent selection pressures can result in genome-wide excess of rare, potentially derived, alleles within a population as compared to a reference neutral population. It was further evident from the genome-wide average DAF (MAF) values of 0.38 (0.26) and 0.32 (0.20), respectively showing that European and African cattle have experienced variable selection pressures. A comparison of chromosome-wise average of DAF and MAF shows a consistently higher selection in European group (Additional file 15: Figure S10). Hence, we further investigated the significant genomic regions of European cattle for their underlying genes in relation to their unique phenotypes. Significant genomic regions were identified on BTA-1, BTA-13, BTA-14 and BTA-16 by CSS (Figure 3-A). These regions have been generally supported by the constituent selection tests and they contain genes of known functional role in several traits of economic importance in European cattle (Figure 4). However, additional genomic regions identified individually by each of the constituent tests – other than common with CSS – did not capture any known genes as candidates of selection signatures (Additional file 13: Table S5).

## Discussion

This study illustrated a new approach, the CSS, for discovery of selection signatures in outbred populations, which combines three commonly used test statistics into a single index. As expected, each of individual tests (*F*_ST_, XP-EHH, ΔDAF/ΔSAF) can distinguish selection from neutrality but targets slightly different characteristics in the genetic polymorphism data that has been shaped by the selection. Hence, there was only partial agreement in the signals of selection (signatures) in these single tests at the candidate loci. Individually, the three tests also identified additional unique significant clusters with no known candidate genes that indicate their lack of sensitivity to localize real selection signature and high false selection signals (Additional file 10: Table S4). Many earlier studies in cattle and sheep reported selection signatures detected based on these individual tests [11-13,18,32,39-42,64].

The strength of CSS is to combine the component signals so that strongly selected regions harbouring a common signal across the constituent test statistics can be identified. The complementary signals from constituent statistics resulted in increased magnitude of CSS at target loci. For example, in dataset D, the highest CSS cluster was found at the candidate gene region whereas, XP-EHH localized 5 Mb upstream and *F*_ST_ and ΔSAF localized their top ranked signals at 4 Mb downstream of this target region (Additional file 7: Figure S4). Overall, our results suggest that the CSS successfully localized candidate gene regions in both species and both traits, thus providing a validation for this method (Figure 1, Table 2).

### Signatures of selection in traits specific groups of cattle and sheep

#### Polled cattle

A cluster of significant SNPs was successfully localized in the candidate region A1 on BTA-1 that flanks the functional mutations in the *POLL* locus for polledness. In addition, there were two significant clusters on BTA-13 and BTA-14. We further investigated dataset A for any additional structure within the subpopulation of selected cattle breeds. In fact, besides the polledness and horn classification, there were also differences in the body size (stature) between the two groups. The polled group (Angus, Belted Galloway, Galloway, Murray Grey, Red Angus, Red Poll and Romosinuano) contains breeds of small to medium body size; whereas, in the horned group all of the breeds were of medium to large size, except Scottish Highland (7% of the horned group samples) which is a small body size breed (Additional file 1: Table S1). Indeed, the significant cluster on BTA-13 is located at the 66 Mb (region A5) which harbours a pair of genes (*UQCC*-*GDF5*) that has been significantly associated with variation in human height [65-67] and body measurement traits in cattle [68]. In European and East Asian human populations, strong signals of recent selection have also been identified near the *GDF5* gene [60]. Similarly, the second most significant additional cluster on BTA-14 (region A7) in dataset A harbours the *PLAG1* and *CHCHD7* genes which have been mapped for stature in cattle and human [17,19,20,48,67,69,70].

#### Double muscle cattle

The highest CSS scores were found in the candidate regions on BTA-2 (region B1) which harbours the functional mutations in *MSTN* gene for double muscling in cattle. In dataset B, several genes of interest were found in four additional clusters at regions B2, B6, and B8. Region B2 contains the *FRYL* gene within the peak at 68.0 Mb position on BTA-6. Significant selection signatures have previously been detected in this region and its flanking gene *PDGFRA*. This gene has been found connected to multiple molecular networks involving β-estradiol and is associated with reproduction in cattle [13]. Region B6 harbours solute carrier family genes, *SLC25A33* and *SLCC45A1* covering the 45.0-46.0 Mb position on BTA-16. This region was reported as carrying highly differentiated loci and extended haplotype homozygosity in multiple breeds [71]. Region B8 contains the *MC1R* gene near the peak at 14.0-15.0 Mb position on BTA-18, where strong selection signatures have previously been identified involving several breeds that have also been used in the present study [15]. The *melanocortin 1 receptor* (*MC1R*) gene is the candidate for coat colour in cattle [13,15,20,72,73].

#### Polled sheep

In polled sheep, the regions with highest CSS scores were on OAR-10 (region C5) near the *RXFP2* gene for polledness. In addition, OAR-13 (region C8) and OAR-25 (region C10) exhibit the two significant peaks at positions 68.0 Mb and 8.0 Mb, respectively. At the peak on OAR-13 the footprints of selection have previously been reported for the *ASIP* gene [11] which controls black and white coat colour in sheep [74]. Selection signatures have also been reported for the cluster on OAR-25 (region C10) but the gene(s) and cause of selection were unknown Kijas et al. [11]. However, the low density *lipoprotein-related protein 4* (*LRP4*) gene, located near this region (C10), controls the inhibitory function on bone growth in human [75], hence, it may have some role in horn formation or it may play some role in the body size by controlling the body bone mass in sheep. Furthermore, this region contains a putative major gene/QTL for wool quality and fibre diameter across a range of breeds [76,77].

#### Double muscle sheep

The genomic region with highest CSS scores was found on OAR-2 (region D4) near the *MSTN* gene for double muscling in Texel sheep breeds. In dataset D, all the additional significant peaks are also on OAR-2 and are in the candidate region or in LD with the candidate gene location spanning a region of almost 10 Mb. These results suggest that a very strong selection pressure would identify a broad genomic region of selection signature, which may limit the power of fine-mapping and identifying the causal mutation in such a resource population. However, the complementary signals from constituent statistics at the target gene notably improved the magnitude of CSS.

Clearly across all traits in both species, composition of the breed panel in which selection signatures are to be detected may give rise to associations with more than one trait, i.e. confounding, which could give rise to spurious signals open to misinterpretation. Hence, independent validation or within-breed linkage studies may be required for further confirmation of such selection signatures.

### Signatures of selection in geographically isolated *Bos taurus*

Strong selection signatures for several economically important traits in European cattle were identified at five genomic regions (Figure 3-A, Figure 4). At the proximal end of BTA-1, the *POLL* locus [53] was identified for the whole group. While the polled phenotypes is not common in all the European breeds, due to the economics of dehorning and increased demand for animal safety acquired in the natural polledness, the *POLL* locus is being introgressed in most of the commercial cattle breeds. Moreover, our results can also be explained as the common haplotypes at the *POLL* locus found to be shared in several polled and horned breeds [47].

On BTA-13, *CDC123* and *CAMK1D* genes have been reported to participate in various functions of pancreatic beta-cell and genetic variants at this locus have been associated to type 2 diabetes susceptibility in human [78]. The associated gene pair has been known for its role in insulin-related metabolic traits [79]. In European cattle, these genes may be involved in several metabolic pathways for sufficient availability of energy for improved growth, production and maintaining body temperature in a temperate environment.

The prominent selection signal at the proximal end of BTA-14 underlies the *DGAT1* gene that has been reported to have a significant role in several traits of dairy and beef production [14,28,80-84]. At another location on BTA-14, the stature (*PLAG1*-*CHCHD7*) genes (as discussed above) were also identified for the European group. The region on BTA-14 has been found to have a significant enrichment for the runs of homozygosity (ROH) – an indicator of strong LD – in the majority of cattle breed types (beef, dairy, English, European etc.) using the SNP data from the 50K and 800K BovineSNP chip assays [46]. Cattle have extensive LD patterns compared to human [85]. LD is likely to be even more extensive in the vicinity of a selective sweep and hence the frequency of selected alleles in these regions is likely to be high, being driven towards fixation [86].

The extreme peak in CSS for European cattle was found near the centre of BTA-16. The existence of a huge CSS can be explained such that several genes at this region have been reported as the candidate of strong selection. Signatures of selection have identified *SLC25A33* and *SLCC45A1* genes for their important role in immunity related to tropical adaptation [71]. Similarly, *PIK3CD* and *SPSB1* genes were also identified under selection respectively linked to immune response and immune regulation in both Angus and Simmental [17]. Signatures of selection have also been found in several breeds for *NMNAT1*[32] and *RERE*[17] genes which have been associated with embryonic growth and reproductive development. At the same location, the *KIF1B* gene was identified under strong selection in Holstein cattle [13]. In addition, another gene, *AGTRAP*, which is located at 1 Mb upstream to the CSS peak, has been identified for dairy production due to its role in mammary glands [15].

Overall, in European cattle, we note that the magnitude of CSS scores corresponded to the diversified and extensive role of underlying candidate genes. This provides further evidence for CSS to capture trait-specific genomic regions as illustrated in validation datasets.

In the African *Bos taurus*, in general, the lack of pronounced selective pressures as compared to the European counterparts has resulted in localizing the significant CSS in non-genic regions or regions harbouring genes of unknown effects (Additional file 13: Table S5). Additional limiting factors such as SNP ascertainment bias and high admixture in African taurine due to excessive crossbreeding with African indicine cattle could also have contributed to the randomly dispersed signatures of selection.

The effects of SNP origin (ascertainment bias) on various estimators of population genetic parameters and some practical methods for correcting them have been discussed elsewhere [87-90]. In cattle, the SNP panel (50K BovineSNP chip) was designed predominately based on the genetic polymorphisms in European breeds [3,17,52] which resulted in low representation of rare variants, thus a lower SNP diversity within some non-European cattle breeds, especially in *Bos indicus* or African *Bos taurus.* The SNP ascertainment bias has been found to have profound effects in the combined analysis of worldwide breeds [3,10,38]. The significant SNPs clustering in composite tests partly depend on the haplotype-based component tests, especially those that are derived from EHH [16,23,38], which can be significantly affected by breeds used to discover SNPs [91].

We adopted a cautious approach by excluding the indicine breeds from the available genotypic data of the African cattle [10,38,49] to minimize diversity within the African cattle group in dataset E. Generally, morphological and genetic data suggest a common origin for African and European taurines [39]. That can be suitable in analyses of multi-breed group comparison, i.e., assuming that both populations are closely related, while differentially selected at a few genomic regions. Nevertheless, phylogenetic investigations have shown early divergence between the African and European taurine cattle and high genetic relatedness between the African taurine and indicine cattle [3,10]. Indicine allelic enrichment in the African taurines Y-chromosome [92] and autosomal SNPs has also suggested a high genetic admixture in several African breeds [10], that could have swept out several taurine-specific genomic regions. Hence, genome-wide high heterogeneity within the African cohort could not help resolve the mapping signature of selection for various candidates of selection; for example, climatic adaptation and resistance to various pathogens in African *Bos taurus*[49,64].

Comprehensive discovery analyses performed within the African breeds are more likely to capture genomic regions that have been targets of selection in those breeds, as the genome-wide scans for selection signatures comparing relatively close populations are least confounded by common biases [93]. Moreover, additional accuracy is also expected by using high-density SNP panels such as the BovineHD SNPchip (800K SNPs) that has been designed to be less sensitive to the ascertainment bias for non-European cattle breeds [94].

Overall, despite the several confounding factors that may have limited the localization of previously known genes in the African cattle, the existence of significant CSS in functionally unknown genes and noncoding regions indicate putative regions under selection. In several species, the non-coding DNA sequences have been predicted for their multiple roles in structural and regulatory mechanism of chromosomes including DNA replication, epigenomic modifications, regulation of transcription and translation. With the knowledge of incomplete genomic annotations and several genes of unknown functions in cattle, the functional importance of noncoding regions under evolutionary selective pressure cannot be underestimated. Functional annotation approaches and resources [95] such as, across species comparison for conserved DNA sequences may help further elucidate the uncharacterized selective sweeps of our results.

### FDR and power of CSS

Considering all the individual regions as independent events of selection in the genome, all the identified genomic regions in validation datasets had a much higher proportion of significant SNPs (*q* < 0.05) as compared to the rest of the genome in each dataset (Figure 2, Table 3). Overall, combining multiple test statistics reduced the false signals of CSS as compared to individual constituent tests (Additional file 10: Table S4). The strategy of grouping the phenotypically alike populations applied in the present study could have further reinforced the selection signals at the common trait’s candidate regions while neutralizing the population specific patterns of diversity elsewhere [15].

The power (sensitivity) of the individual methods to discriminate between true positives (due to directional selection) and false positives (due to the forces other than selection) is a critical factor in the choice of selection tests. A combination of multiple statistics is expected to improve the power of composite statistics by complementing the detectability of positive selection by individual tests [96], e.g., the haplotype-based tests may be affected due to the distribution of recombination hotspots across the genome [31]. Haplotypes, on the other hand, being patterns of multiple SNPs, are less sensitive to ascertainment schemes of the genome-wide panels of SNPs. SNP-based tests localize in unknown and non-genic regions more frequently and are less specific as compared to haplotype estimates as shown in (Additional file 10: Table S4) and Qanbari et al. [32]. CSS combines multiple characteristics of the genetic diversity from the single locus polymorphisms and haplotype patterns which makes it less sensitive to the confounding effects of demography and recombination [97,98]. However, CSS being a composite of SNP and haplotype-based test statistic, can still be sensitive to SNP density, SNP ascertainment bias and the extent and variation of LD across the genome.

The power of most studies of genome-wide selection scans is low because of the small sample sizes, SNP density, SNP ascertainment scheme and the test statistics used. Panels of outbred populations consisting of multiple breeds can be used to increase the sample size and to enhance the power of CSS. A large number of samples genotyped with various SNP panels are becoming available in many species. These data can be combined using imputation strategies [99] to increase the power of CSS.

It is noteworthy to mention that without simulations, qualitative evaluation of gain in power of CSS is not possible for comparison with the constituent tests. Similarly, a direct comparison of CSS with the previously published methods of combining multiple statistics [16,23,31,33,34,100] requires simulation data from robust models to depict the underlying dynamics of the population of interest along with powerful computational tools for permutation iterations [15]. Such comparisons are difficult for a real dataset where it is almost impossible to subset contrasting populations for a single event of selection. However, successful and improved localization of candidate genes in cattle and sheep, by simply combing rank distributions of constituent tests, indicates the power of CSS. Moreover, CSS can incorporate additional test statistics to add power for localizing the selection signature. The choice of additional tests to incorporate complementary evidence may be based on their unique power under various assumptions of selection, availability of data information (phasing of ancestral and derived alleles) and *a priori* assumptions about the dynamics of populations of interest. Established selection tests, such as the across-population Rsb [30] test and within-population estimates of positive selection including integrated haplotype scores (iHS) [60], haplotype allelic count statistics called Svd [97] and composite log likelihood (CLL) [15], can also be included in the CSS computation. However, combining too many selection tests of similar specificity might bias the CSS scores towards those characteristics of related tests. This bias may be misleading when interpretations are made to generalize the contribution of all the component test statistics. Care is thus required to select constituent tests of CSS.

CSS is expected to be successful in identifying the candidate genes for complex networks and selection events e.g., domestication, adaptation and production traits. Complex traits are usually controlled by a very large number of loci of small effects; consequently, selective pressures on their causal mutations drive a very slow change in the allele frequencies. This makes it more challenging to discover such genetic variants of small effects. Comprehensive phenotypic records and robust trait-wise classification are required to efficiently characterize complex traits under selection. CSS can be further tuned with additional selection tests appropriate to distinguish genomic regions under selection for complex traits. To robustly map positive selection for complex traits, some specialized tests, such as birth date selection mapping [101] designed to identify small changes in the allele frequency due to selection of polygenic traits can be appropriate. The biological functions underlying polygenic inheritance are controlled by the interactions between large networks of genes. Selective pressures depend on the degree of contribution and the position of genes in the network [102]. The evolutionary properties of the complex traits can be captured by exploring gene networks for the genes under the selective sweeps. Overall, using CSS along with GWAS [34], QTL mapping [100] and approaches including gene pathways [103] can elucidate the mechanism underlying diversity in complex traits.

## Conclusion

We developed a method, composite selection signals (CSS), which appears to be efficient in identifying selection signatures for traits and genes that have evolved rapidly under various selection pressures. It is a very robust method for detecting selection signatures, as it does not depend on any distributional assumptions (normality) of the constituent test statistics, and additional test statistics can easily be included, if they become available. The existence of strong signals linked to known candidate genes, even in the absence of any casual SNP in the genotype data, validates the utility of the breed grouping strategy and methodology for deriving composite selection signals. In addition, estimates of FDR also provide clear evidence that any cluster of significant SNPs captured by CSS is highly likely to contain a strong candidate (gene or variant) of positive selection.

The majority of significant peaks outside the candidate regions in validation subsets were linked to various additional phenotypic classifications of cattle and sheep cohorts. For example, implementation of CSS identified *UQCC*-*GDF5* as the plausible candidate genes for stature which have known effects on development and skeletal growth. Our results also replicated the previously reported candidate locus containing *PLAG1*-*CHCHD7* genes for stature in cattle. Other notable secondary phenotypes include; coat colour, reproduction, bone growth and multiple functioning transporters of the solute carrier family of genes.

In European cattle, the historical impacts of long-term selection pressures for economically important traits were identified for polledness, adaptation, metabolism, growth rate, stature, immunity, reproduction and several candidate genes related to dairy and beef production.

The presence of spurious selection signals is much lower in CSS as compared to individual constituent tests due to the unique signals of each constituent selection test are reduced while combining multiple test statistics. Taken together, CSS provides an improvement for the predictions of positive selection and demonstrates that probing the multiple pieces of evidence for positive selection can provide important insights into understanding trait-specific gene evolution.

## Data availability

R scripts and high quality images are available from the corresponding author on request.

## Abbreviations

CSS: Composite selection signals; SNP: Single nucleotide polymorphism; bp: Base pair; Mb: Mega-base; BTA: *Bos taurus* (cattle) autosome; OAR: *Ovis aries* (sheep) autosome; MAF: Minor allele frequency; DAF: Derived allele frequency; ∆DAF: Change in DAF; ∆SAF: Change in the selected allele frequency; FST: Fixation index; LRH: Long range haplotype; EHH: Extended haplotype homozygosity; XP-EHH: Across population EHH; iHS: Integrated haplotype scores; IBS: Identical by state; LD: Linkage disequilibrium; ROH: Runs of homozygosity; FDR: False discovery rate; CDF: Cumulative distribution function; CLL: Composite log likelihood; CMS: Composite of multiple signals; Meta-SS: Meta-analysis of selection signals.

## Competing interests

The authors declare that they have no competing interests.

## Authors’ contributions

IASR conceived the idea, designed the study, performed the analyses and wrote the manuscript. MSK, PCT and HWR supervised the study, contributed to interpret the results and edit the manuscript. MSK contributed in the design, data acquisition, QC, data analysis and preparation of the manuscript. PCT contributed in the statistical approaches to establish the new method, R scripts and critically reviewed the manuscript. HWR is the principal supervisor, group leader, provided the research directions and overall guidance. All authors read and approved the final manuscript.

## Supplementary Material

Additional file 1: Table S1 The information about the breeds, animals and phenotype categories of cattle samples. Click here for file

Additional file 2: Table S2 The information about the breeds, animals and phenotype categories of sheep samples. Click here for file

Additional file 3: Table S3 Chromosome wise information regarding genotyping data of cattle and sheep. Click here for file

Additional file 4: Figure S1 Manhattan plots of SNP-wise scores for each selection test statistics (A: CSS, B: *F*_ST_, C: XP-EHH, D: ΔDAF) for polled cattle (dataset A). Gray dots in the background show raw scores and blue and orange dots in the foreground show smooth scores, averaged over SNPs within 1 Mb sliding windows. Red dotted lines indicate a threshold of top 0.1 percentile of the genome-wide smoothed scores for each of the selection test statistics. Red square dots in each plot show the genome-wide highest raw signals. Click here for file

Additional file 5: Figure S2 Manhattan plots of SNP-wise scores for each selection test statistics (A: CSS, B: *F*_ST_, C: XP-EHH, D: ΔDAF) for double muscle cattle (dataset B). Gray dots in the background show raw scores and blue and orange dots in the foreground show smooth scores, averaged over SNPs within 1 Mb sliding windows. Red dotted lines indicate a threshold of top 0.1 percentile of the genome-wide smoothed scores for each of the selection test statistics. Red square dots in each plot show the genome-wide highest raw signals. The square dots are in dark brown colour in B plot as the highest *F*_ST_ signals is more than 3 Mb upstream from the known candidate region on chromosome 2. Click here for file

Additional file 6: Figure S3 Manhattan plots of SNP-wise scores for each selection test statistics (A: CSS, B: *F*_ST_, C: XP-EHH, D: ΔSAF) for polled sheep (dataset C). Gray dots in the background show raw scores and blue and orange dots in the foreground show smooth scores, averaged over SNPs within 1 Mb sliding windows. Red dotted lines indicate a threshold of top 0.1 percentile of the genome-wide smoothed scores for each of the selection test statistics. Red square dots in each plot show the genome-wide highest raw signals. Click here for file

Additional file 7: Figure S4 Manhattan plots of SNP-wise scores for each selection test statistics (A: CSS, B: *F*_ST_, C: XP-EHH, D: ΔSAF) for double muscle sheep (dataset D). Gray dots in the background show raw scores and blue and orange dots in the foreground show smooth scores, averaged over SNPs within 1 Mb sliding windows. Red dotted lines indicate a threshold of top 0.1 percentile of the genome-wide smoothed scores for each of the selection test statistics. Red square dots in each plot show the genome-wide highest raw signals. The square dots are in dark brown colour in C and D plots where the highest XPEHH and ΔSAF signals are more than 3 Mb upstream and downstream, respectively, from the known candidate region on chromosome 2. Click here for file

Additional file 8: Figure S5 Distribution of the number of SNPs in 1 Mb sliding windows in cattle (A) and sheep (B). Bars in A and B indicate the frequency of sliding windows containing various number of SNPs out of the genome-wide distribution, i.e., 38,610 SNPs of cattle and 47,502 SNPs of sheep data, respectively (details in Table 1, S3). The bars in red (black) colours show the mean ≈ median (mode) numbers as 17 (18) and 19 (20) of SNPs for cattle and sheep data, respectively. Click here for file

Additional file 9: Figure S6 Genome-wide pairs plots (lower diagonals), histograms (diagonals) and correlations (upper diagonals) for constituent (XP-EHH, ΔSAF, *F*_ST_) and composite selection signals (CSS) for polled (A), double muscle (B), cattle polledness (C) and double muscling (D) in sheep. Click here for file

Additional file 10: Table S4 Complete list of genomic regions and genes harbouring significant SNPs identified by four tests in four cohorts of cattle and sheep data. Cluster of minimum three significant SNPs within a window spanning 1 Mb genomic locations centred on a core SNP above the threshold (top 0.1%) in multiple tests (smoothed statistics) are reported and are compared with each other. Click here for file

Additional file 11: Figure S7 Histograms of Mean *Z*, raw *p*-value and calibrated *p*-values distributions of the CSS: Histograms (top to bottom in each column) for polled cattle (column 1, red), double muscle cattle (column 2, green), polled sheep (column 3, purple) and double muscle sheep (column 4, blue). Click here for file

Additional file 12: Figure S8 False discovery rate (FDR) against *p*-values: *q*-values were calculated from the calibrated *p*-values. Vertical dotted (……) and dashed (−−−−−) lines indicate calibrated *p*-values at 0.01 and 0.05, respectively. Horizontal dotted and dashed lines indicate *q*-values (FDR) at 0.05 and 0.1, respectively. Click here for file

Additional file 13: Table S5 Selection signatures in European and African *Bos taurus* cattle populations. Complete list of selection signatures identified by composite (CSS) and constituent (XPEHH, FST, ΔDAF) selection tests. Click here for file

Additional file 14: Figure S9 Genome-wide comparison of using SNP genotype data from all breeds (Total: 46 breeds and N = 847), breeds with minimum 10 samples (Total: 26 breeds and N = 753) and breeds with minimum 20 samples (Total: 20 breeds and N = 652) for computing CSS (A), XP-EHH (B), ΔDAF (C) and *F*_*ST*_ (D) for European *Bos taurus* cattle. Click here for file

Additional file 15: Figure S10 Chromosome-wise comparison of average derived allele frequencies (A) and average minor allele frequencies (B) between European and African *Bos taurus* cattle breeds. Click here for file
