# Supplementary material for: Composite selection signals can localize the trait specific genomic regions in multi-breed populations of cattle and sheep
Source: BMC Genet. 2014 Mar 17;15:34. doi: 10.1186/1471-2156-15-34 (PMC4101850; doi:10.1186/1471-2156-15-34)
Supplement: Additional file 1: Table S1 — The information about the breeds, animals and phenotype categories of cattle samples. [file 1471-2156-15-34-S1.pdf]

Additional file 1

Table S1. The information about the breeds, animals and phenotype categories of cattle sample

No	*Cattle Breeds*	*Country of origin (and sampling, if different)*	*Phenotype Categories*	*Body size*	*Geographic Categories*	*Sample Size*
1	Angus	Britain (USA and NZ)	Poll, Normal muscle	Small	Europe	44
2	Red Angus	Britain (USA)	Poll, Normal muscle	Small	Europe	15
3	Red Poll	Britain (USA)	Poll	Small	Europe	5
4	Belted Galloway	Britain	Poll	Medium	Europe	4
5	Galloway	Britain	Poll	Medium	Europe	4
6	Murray Grey	Australia (USA)	Poll	Medium	-	5
7	Romosinuano	New World Spanish	Poll, Normal muscle	Small	-	8
8	Hereford	Britain (USA, NZ)	Normal muscle	Medium	Europe	31
9	Limousin	France (USA)	Normal muscle	Medium	Europe	35
10	Charolais	France (Britain, USA)	Normal muscle	Large	Europe	55
11	Lincoln Red	Britain (USA)	Normal muscle	Large	Europe	9
12	Simmental	Switzerland (USA)	Normal muscle	Large	Europe	10
13	Scottish Highland	Britain (and USA)	Horn, Normal muscle	Small	Europe	9
14	Texas Longhorn	New World Spanish	Horn, Normal muscle	Medium	-	10
15	Baoule	Burkina Faso	Horn, Normal muscle	Medium	Africa	29
16	Chianina	Italy (USA)	Horn, Normal muscle	Large	Europe	8
17	Maine Anjou	France (and USA)	Horn, Normal muscle	Large	Europe	21
18	Romagnola	Italy	Horn, Normal muscle	Large	Europe	24
19	Piedmontese	Italy	Horn, Double muscle	Medium	Europe	26
20	Parthenais (Maraichine)	France	Double muscle	Large	Europe	19
21	Belgian Blue	Belgium (USA)	Double muscle	Large	Europe	4
22	Borgou	Benin	-	-	Africa	30
23	Lagune	Benin	-	-	Africa	30
24	NDama	West Africa (Burkina Faso, Guinea and Gambia)	-	-	Africa	61
25	Oulmès Zaer	Morocco	-	-	Africa	26
26	Sheko	East Africa (Ethiopia)	-	-	Africa	20
27	Somba	Togo	-	-	Africa	30
28	Abondance	France	-	-	Europe	22
29	Aubrac	France	-	-	Europe	22
30	Blonde dAquitaine	France (USA)	-	-	Europe	5
31	Bretonne Black Pied	France	-	-	Europe	18
32	Brown Swiss	Switzerland (USA)	-	-	Europe	41
33	Devon	Britain	-	-	Europe	4
34	Dexter	Ireland (Britain)	-	-	Europe	4

Additional file 1

No	Cattle Breeds	Country of origin (and sampling, if different)	Phenotype Categories	Body size	Geographic Categories	Sample Size
35	Finnish Ayrshire	Finland	-	-	Europe	10
36	French Red Pied Lowland	France	-	-	Europe	22
37	Gascon	France	-	-	Europe	22
38	Gelbvieh	Germany (USA)	-	-	Europe	8
39	Guernsey	Channel Islands (UK, USA)	-	-	Europe	21
40	Holstein	Netherlands (France, NZ and USA)	-	-	Europe	80
41	Jersey	Channel Islands (France, USA and NZ)	-	-	Europe	49
42	Kerry	Ireland (Britain)	-	-	Europe	3
43	Longhorn	Britain	-	-	Europe	3
44	Marchigiana	Italy (USA)	-	-	Europe	5
45	Montbeliarde	France	-	-	Europe	35
46	Normande	France	-	-	Europe	31
47	Norwegian Red	Norway	-	-	Europe	21
48	Pinzgauer	Austria (USA)	-	-	Europe	5
49	Salers	France (and USA)	-	-	Europe	27
50	Shorthorn	Britain (USA)	-	-	Europe	10
51	South Devon	Britain	-	-	Europe	4
52	Sussex	Britain	-	-	Europe	4
53	Tarine Tarentaise	France (and USA)	-	-	Europe	23
54	Vosgienne Vosges	France	-	-	Europe	19
55	Welsh Black	Britain	-	-	Europe	2
56	White Park	Britain	-	-	Europe	4

NZ: New Zealand, **UK**: United Kingdom (Britain), **USA**: United States of America
