# Supplementary material for: Composite selection signals can localize the trait specific genomic regions in multi-breed populations of cattle and sheep
Source: BMC Genet. 2014 Mar 17;15:34. doi: 10.1186/1471-2156-15-34 (PMC4101850; doi:10.1186/1471-2156-15-34)
Supplement: Additional file 2: Table S2 — The information about the breeds, animals and phenotype categories of sheep samples. [file 1471-2156-15-34-S2.pdf]

Additional file 2

Table S2. The information about the breeds, animals and phenotype categories of sheep samples

No.	*Sheep Breeds*	*Phenotype Categories*	*Sample Size*
1	Afec Assaf	Poll, Normal muscle	24
2	African Dorper	Poll, Normal muscle	20
3	African White Dorper	Poll, Normal muscle	6
4	Afshari	Poll, Normal muscle	37
5	Altamurana	Horn, Normal muscle	24
6	Arawapa	Horn, Normal muscle	37
7	Australian Coopworth	Poll, Normal muscle	19
8	Australian Industry Merino	Horn, Normal muscle	86
9	Australian Merino	Horn, Normal muscle	50
10	Australian Poll Dorset	Poll, Normal muscle	105
11	Australian Poll Merino	Poll, Normal muscle	98
12	Australian Suffolk	Poll, Normal muscle	108
13	Bangladeshi BGE	Horn, Normal muscle	24
14	Bangladeshi Garole	Horn, Normal muscle	24
15	Barbados Black Belly	Poll, Normal muscle	24
16	Border Leicester	Poll, Normal muscle	47
17	Boreray	Horn, Normal muscle	17
18	Brazilian Creole	Horn, Normal muscle	23
19	Castellana	Horn, Normal muscle	23
20	Changthangi	Horn, Normal muscle	29
21	Churra	Horn, Normal muscle	119
22	Comisana	Poll, Normal muscle	24
23	Cyprus Fat Tail	Horn, Normal muscle	30
24	Deccani	Poll, Normal muscle	23
25	Dorset Horn	Horn, Normal muscle	21
26	East Friesian Brown	Poll, Normal muscle	39
27	East Friesian White	Poll, Normal muscle	9
28	Egyptian Barki	Horn, Normal muscle	13
29	Engadine Red Sheep	Poll, Normal muscle	24
30	Ethiopian Menz	Horn, Normal muscle	34
31	Finnsheep	Poll, Normal muscle	99
32	Galway	Poll, Normal muscle	49
33	Garut	Horn, Normal muscle	22
34	German Texel	Poll, Double muscle	46
35	Gulf Coast Native	Horn, Normal muscle	93
36	Icelandic	Horn, Normal muscle	16
37	Improved Awassi	Poll, Normal muscle	23

Additional file 2

38	Indian Garole	Horn, Normal muscle	26
39	Irish Suffolk	Poll, Normal muscle	49
40	Karakas	Poll, Normal muscle	17
41	Leccese	Horn, Normal muscle	24
42	Local Awassi	Horn, Normal muscle	24
43	Macarthur Merino	Horn, Normal muscle	10
44	Meat Lacaune	Poll, Normal muscle	78
45	Merinolandschaf	Poll, Normal muscle	24
46	Milk Lacaune	Poll, Normal muscle	103
47	Moghani	Poll, Normal muscle	34
48	Morada Nova	Poll, Normal muscle	22
49	Namaqua Afrikaner	Horn, Normal muscle	12
50	New Zealand Romney	Poll, Normal muscle	24
51	New Zealand Texel	Poll, Double muscle	23
52	Norduz	Horn, Normal muscle	20
53	Ojalada	Horn, Normal muscle	24
54	Old Norwegian spaelsau	Horn, Normal muscle	15
55	Qezel	Poll, Normal muscle	35
56	Rambouillet	Horn, Normal muscle	101
57	Rasaaragonesa	Poll, Normal muscle	22
58	Red Maasai	Horn, Normal muscle	44
59	Ronderib Afrikaner	Horn, Normal muscle	17
60	Sakiz	Horn, Normal muscle	22
61	SantaInes	Poll, Normal muscle	47
62	Sardinian Ancestral Black	Horn, Normal muscle	20
63	Scottish Blackface	Horn, Normal muscle	56
64	Scottish Texel	Poll, Double muscle	80
65	Soay	Horn, Normal muscle	110
66	Spael-coloured	Poll, Normal muscle	3
67	Spael-white	Poll, Normal muscle	32
68	Sumatra	Normal muscle	24
69	SwissBlack-Brown Mountain Sheep	Poll, Normal muscle	24
70	Swiss Mirror Sheep	Poll, Normal muscle	24
71	Swiss White Alpine Sheep	Poll, Normal muscle	24
72	Tibetan	Horn, Normal muscle	37
73	Valais Red Sheep	Horn, Normal muscle	23
74	Wiltshire	Horn, Normal muscle	20
