# Supplementary material for: Composite selection signals can localize the trait specific genomic regions in multi-breed populations of cattle and sheep
Source: BMC Genet. 2014 Mar 17;15:34. doi: 10.1186/1471-2156-15-34 (PMC4101850; doi:10.1186/1471-2156-15-34)
Supplement: Additional file 3: Table S3 — Chromosome wise information regarding genotyping data of cattle and sheep. [file 1471-2156-15-34-S3.pdf]

Additional file 3

Table S3. Chromosome wise information regarding genotyping data of cattle and sheep

Chromosome	CATTLE					SHEEP			
	Length [a] (Mbp)	SNPs (N)	Ancestral [b]	Density (bp) [c]		Length [a] (Mbp)	SNPs (N)	Density (bp) [c]	
				Mean	Maximum			Mean	Maximum
**1**	159	2555	2550	61,874	1,160,151	300	5336	56,156	941,607
**2**	138	2062	2058	66,108	1,715,336	264	5009	52,537	984,461
**3**	122	1952	1947	62,058	1,610,036	243	4535	53,497	1,335,673
**4**	122	1928	1925	62,474	624,584	128	2463	51,629	557,472
**5**	122	1608	1596	74,398	988,849	117	2147	54,035	842,189
**6**	120	1909	1906	62,391	1,601,814	130	2358	54,736	2,965,708
**7**	114	1671	1667	67,285	3,404,489	109	2029	53,585	1,053,010
**8**	114	1836	1830	61,586	650,324	98	1870	52,317	463,761
**9**	107	1572	1566	66,537	932,818	101	1931	52,223	789,815
**10**	105	1661	1658	62,201	3,259,342	95	1676	56,177	3,487,146
**11**	108	1697	1689	63,075	799,678	67	1103	60,675	477,632
**12**	92	1226	1225	72,793	4,498,320	87	1546	55,684	1,167,568
**13**	85	1348	1344	62,212	1,958,406	90	1556	57,146	902,996
**14**	86	1258	1251	66,134	3,618,794	70	1054	65,289	1,567,687
**15**	86	1239	1236	67,665	1,072,079	91	1530	58,755	1,855,199
**16**	83	1203	1199	66,632	2,397,240	78	1416	54,472	424,043
**17**	76	1200	1196	62,458	839,722	79	1296	60,573	562,710
**18**	67	966	962	67,447	1,106,253	73	1287	55,902	538,746
**19**	65	1013	1011	62,086	544,787	65	1137	57,037	509,668
**20**	73	1200	1194	59,712	559,097	56	1015	54,770	1,069,142
**21**	73	1032	1027	66,222	502,239	56	799	69,095	2,422,034
**22**	62	1004	1002	60,808	465,568	56	988	55,743	2,257,615
**23**	54	793	792	65,311	1,145,617	67	1015	65,377	715,163
**24**	64	964	963	64,447	577,231	45	669	66,283	343,630
**25**	44	703	702	60,893	520,202	49	903	53,262	589,556
**26**	53	802	800	63,560	760,661	51	834	59,809	1,691,855
**27**	46	717	713	63,229	1,341,874	-	-	-	-
**28**	47	700	696	66,084	2,145,618	-	-	-	-
**29**	53	791	789	63,724	901,776	-	-	-	-
**Total**	**2540**	**38610**	**38494**			**2665**	**47502**		
**(Average)**				**(64,531)**	**(1,438,031)**			**(51,268)**	**(1,052,279)**

[a] Chromosomal length according to UMD3.1 Bovine genome assembly and OAR-V1 Ovine genome assembly respectively
[b] Number of SNPs with their known Ancestral and Derived alleles (cattle only)
[c] Average and maximum distance between consecutive SNPs on each autosome
