# Supplementary material for: Composite selection signals can localize the trait specific genomic regions in multi-breed populations of cattle and sheep
Source: BMC Genet. 2014 Mar 17;15:34. doi: 10.1186/1471-2156-15-34 (PMC4101850; doi:10.1186/1471-2156-15-34)
Supplement: Additional file 4: Figure S1 — Manhattan plots of SNP-wise scores for each selection test statistics (A: CSS, B: FST, C: XP-EHH, D: ΔDAF) for polled cattle (dataset A). Gray dots in the background show raw scores and blue and orange dots in the foreground show smooth scores, averaged over SNPs within 1 Mb sliding windows. Red dotted lines indicate a threshold of top 0.1 percentile of the genome-wide smoothed scores for each of the selection test statistics. Red square dots in each plot show the genome-wide highest raw signals. [file 1471-2156-15-34-S4.pdf]

Additional file 4

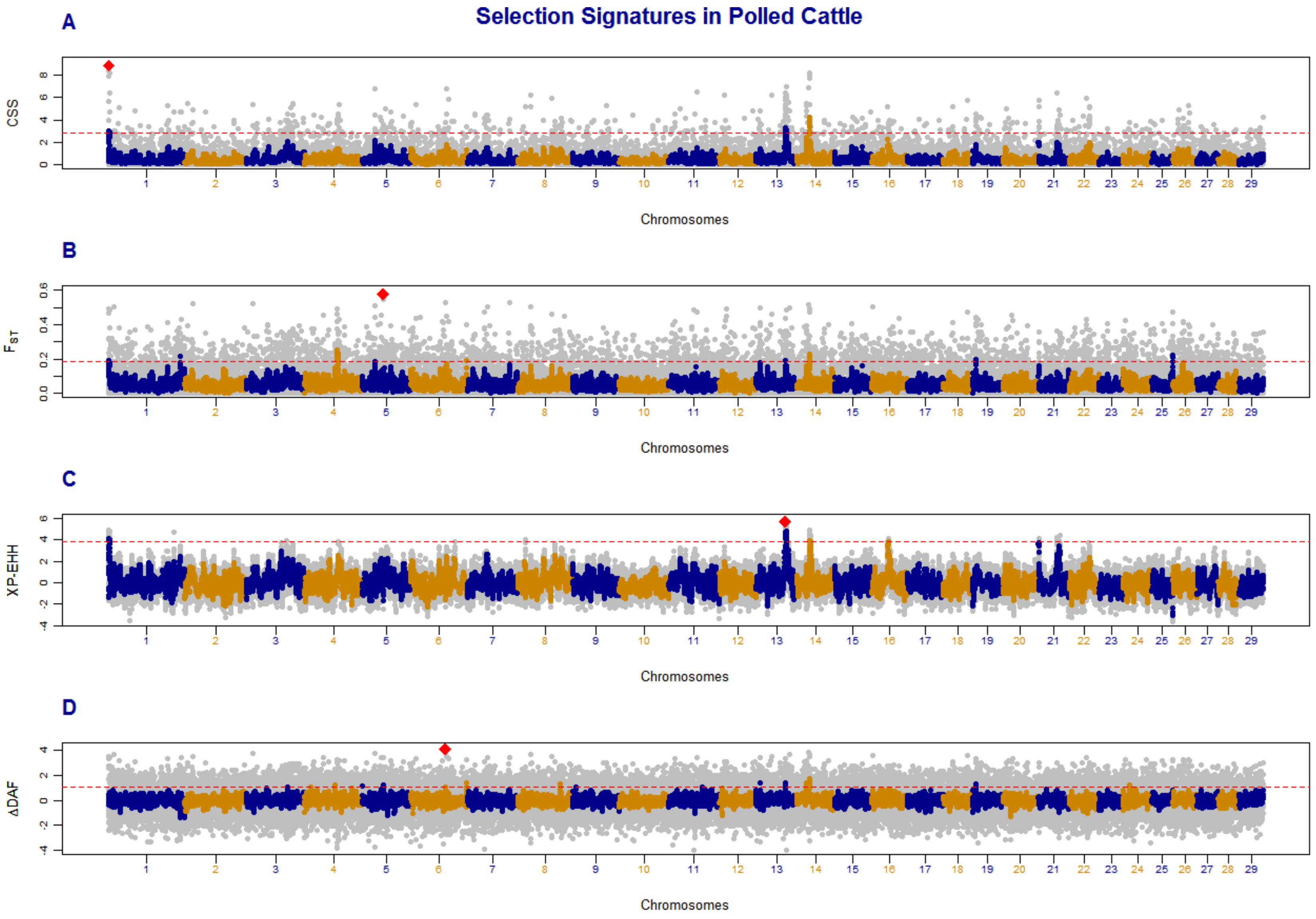

Figure S1. Manhattan plots of SNP-wise scores for each selection test statistics (A: CSS, B: F_{ST}, C: XP-EHH, D: ΔDAF) for polled cattle (dataset A). Gray dots in the background show raw scores and blue and orange dots in the foreground show smooth scores, averaged over SNPs within 1 Mb sliding windows. Red dotted lines indicate a threshold of top 0.1 percentile of the genome-wide smoothed scores for each of the selection test statistics. Red square dots in each plot show the genome-wide highest raw signals.
