# Supplementary material for: Composite selection signals can localize the trait specific genomic regions in multi-breed populations of cattle and sheep
Source: BMC Genet. 2014 Mar 17;15:34. doi: 10.1186/1471-2156-15-34 (PMC4101850; doi:10.1186/1471-2156-15-34)
Supplement: Additional file 6: Figure S3 — Manhattan plots of SNP-wise scores for each selection test statistics (A: CSS, B: FST, C: XP-EHH, D: ΔSAF) for polled sheep (dataset C). Gray dots in the background show raw scores and blue and orange dots in the foreground show smooth scores, averaged over SNPs within 1 Mb sliding windows. Red dotted lines indicate a threshold of top 0.1 percentile of the genome-wide smoothed scores for each of the selection test statistics. Red square dots in each plot show the genome-wide highest raw signals. [file 1471-2156-15-34-S6.pdf]

Additional file 6

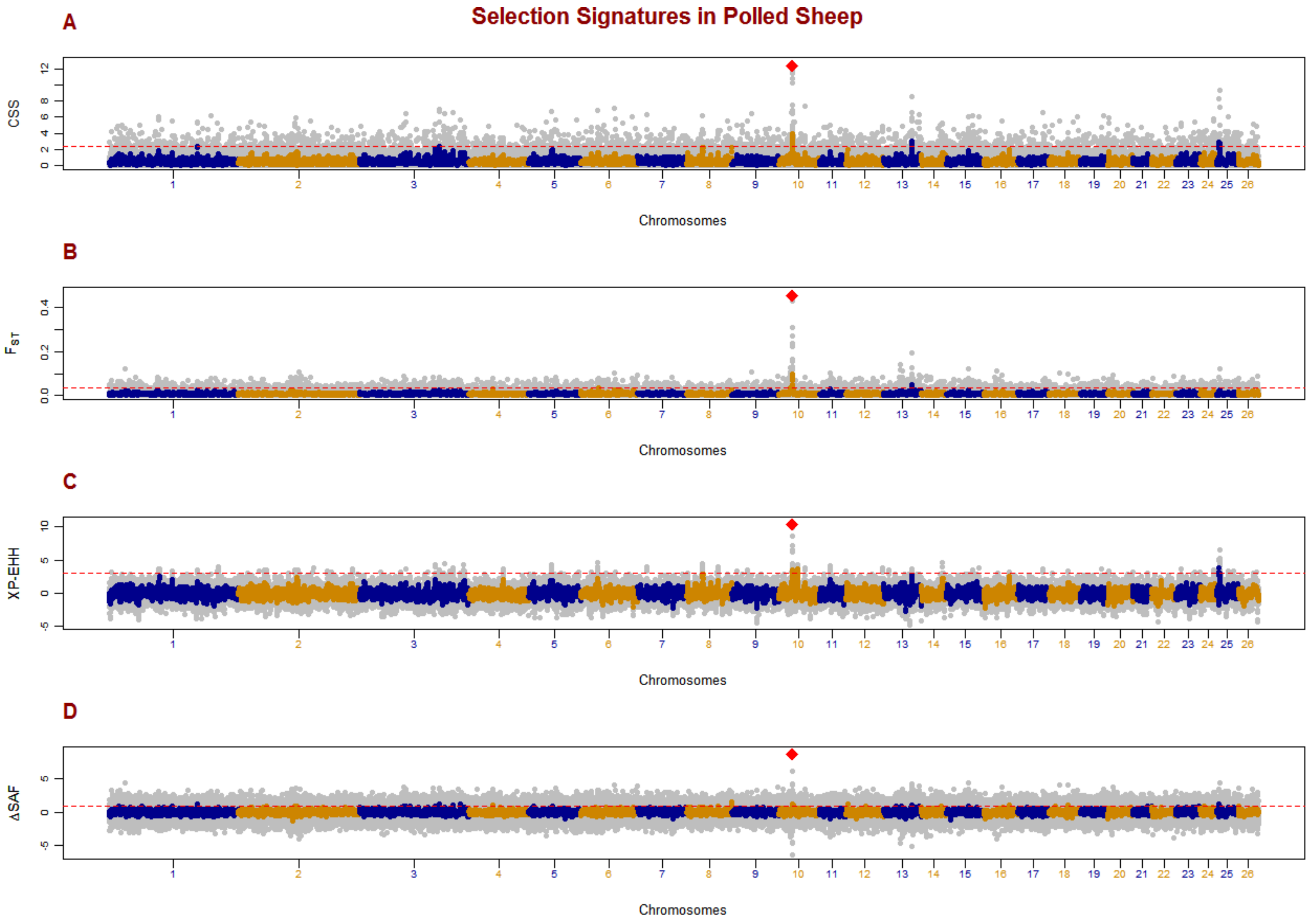


**Figure S3. Manhattan plots of SNP-wise scores for each selection test statistics (A: CSS, B: $F_{ST}$, C: XP-EHH, D: ΔSAF) for polled sheep (dataset C).** Gray dots in the background show raw scores and blue and orange dots in the foreground show smooth scores, averaged over SNPs within 1 Mb sliding windows. Red dotted lines indicate a threshold of top 0.1 percentile of the genome-wide smoothed scores for each of the selection test statistics. Red square dots in each plot show the genome-wide highest raw signals.
