# Supplementary material for: Composite selection signals can localize the trait specific genomic regions in multi-breed populations of cattle and sheep
Source: BMC Genet. 2014 Mar 17;15:34. doi: 10.1186/1471-2156-15-34 (PMC4101850; doi:10.1186/1471-2156-15-34)
Supplement: Additional file 7: Figure S4 — Manhattan plots of SNP-wise scores for each selection test statistics (A: CSS, B: FST, C: XP-EHH, D: ΔSAF) for double muscle sheep (dataset D). Gray dots in the background show raw scores and blue and orange dots in the foreground show smooth scores, averaged over SNPs within 1 Mb sliding windows. Red dotted lines indicate a threshold of top 0.1 percentile of the genome-wide smoothed scores for each of the selection test statistics. Red square dots in each plot show the genome-wide highest raw signals. The square dots are in dark brown colour in C and D plots where the highest XPEHH and ΔSAF signals are more than 3 Mb upstream and downstream, respectively, from the known candidate region on chromosome 2. [file 1471-2156-15-34-S7.pdf]

Additional file 7

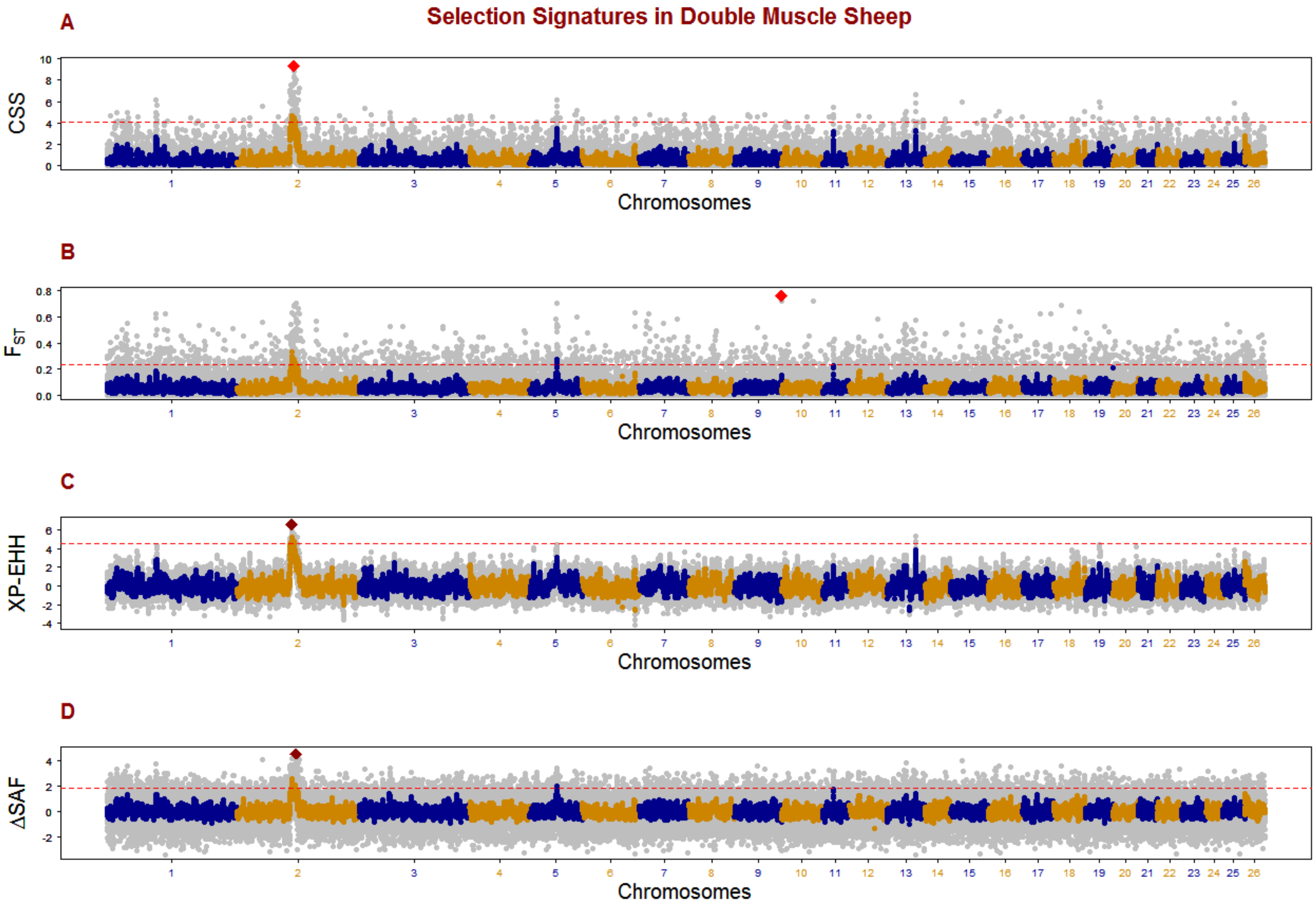

Figure S4. Manhattan plots of SNP-wise scores for each selection test statistics (A: CSS, B: F_{ST}, C: XP-EHH, D: ΔSAF) for double muscle sheep (dataset D). Gray dots in the background show raw scores and blue and orange dots in the foreground show smooth scores, averaged over SNPs within 1 Mb sliding windows. Red dotted lines indicate a threshold of top 0.1 percentile of the genome-wide smoothed scores for each of the selection test statistics. Red square dots in each plot show the genome-wide highest raw signals. The square dots are in dark brown colour in C and D plots where the highest XPEHH and ΔSAF signals are more than 3 Mb upstream and downstream, respectively, from the known candidate region on chromosome 2.
