# Supplementary material for: Composite selection signals can localize the trait specific genomic regions in multi-breed populations of cattle and sheep
Source: BMC Genet. 2014 Mar 17;15:34. doi: 10.1186/1471-2156-15-34 (PMC4101850; doi:10.1186/1471-2156-15-34)
Supplement: Additional file 8: Figure S5 — Distribution of the number of SNPs in 1 Mb sliding windows in cattle (A) and sheep (B). Bars in A and B indicate the frequency of sliding windows containing various number of SNPs out of the genome-wide distribution, i.e., 38,610 SNPs of cattle and 47,502 SNPs of sheep data, respectively (details in Table 1, S3). The bars in red (black) colours show the mean ≈ median (mode) numbers as 17 (18) and 19 (20) of SNPs for cattle and sheep data, respectively. [file 1471-2156-15-34-S8.pdf]

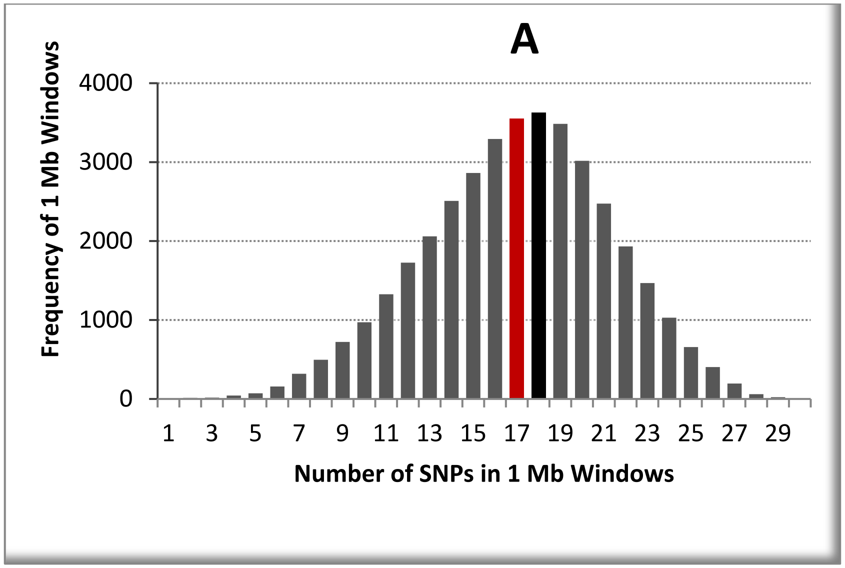


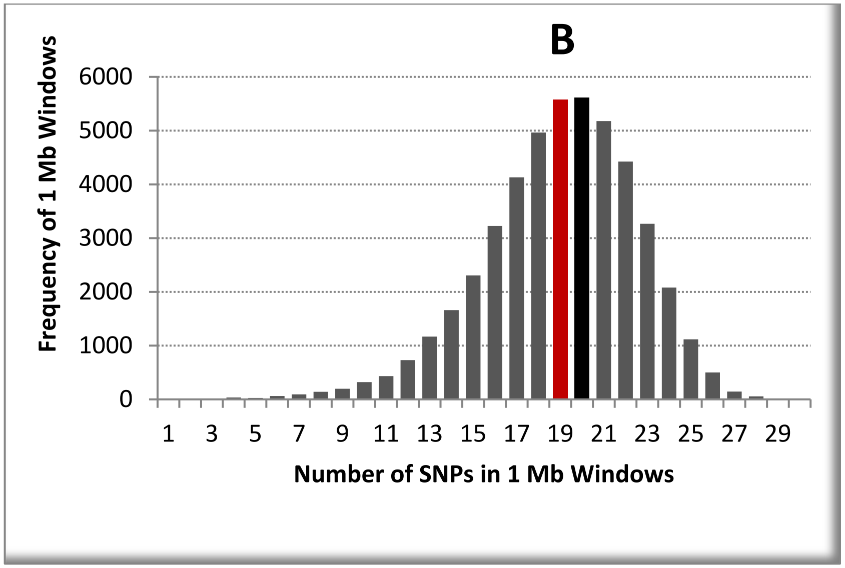


**Figure S5. Distribution of the number of SNPs in 1 Mb sliding windows in cattle (A) and sheep (B).** Bars in A and B indicate the frequency of sliding windows containing various number of SNPs out of the genome-wide distribution, i.e., 38,610 SNPs of cattle and 47,502 SNPs of sheep data, respectively (details in Table 1, S3). The bars in red (black) colours show the mean ≈ median (mode) numbers as 17 (18) and 19 (20) of SNPs for cattle and sheep data, respectively.
