# Supplementary material for: Composite selection signals can localize the trait specific genomic regions in multi-breed populations of cattle and sheep
Source: BMC Genet. 2014 Mar 17;15:34. doi: 10.1186/1471-2156-15-34 (PMC4101850; doi:10.1186/1471-2156-15-34)
Supplement: Additional file 9: Figure S6 — Genome-wide pairs plots (lower diagonals), histograms (diagonals) and correlations (upper diagonals) for constituent (XP-EHH, ΔSAF, FST) and composite selection signals (CSS) for polled (A), double muscle (B), cattle polledness (C) and double muscling (D) in sheep. [file 1471-2156-15-34-S9.pdf]

Additional file 9

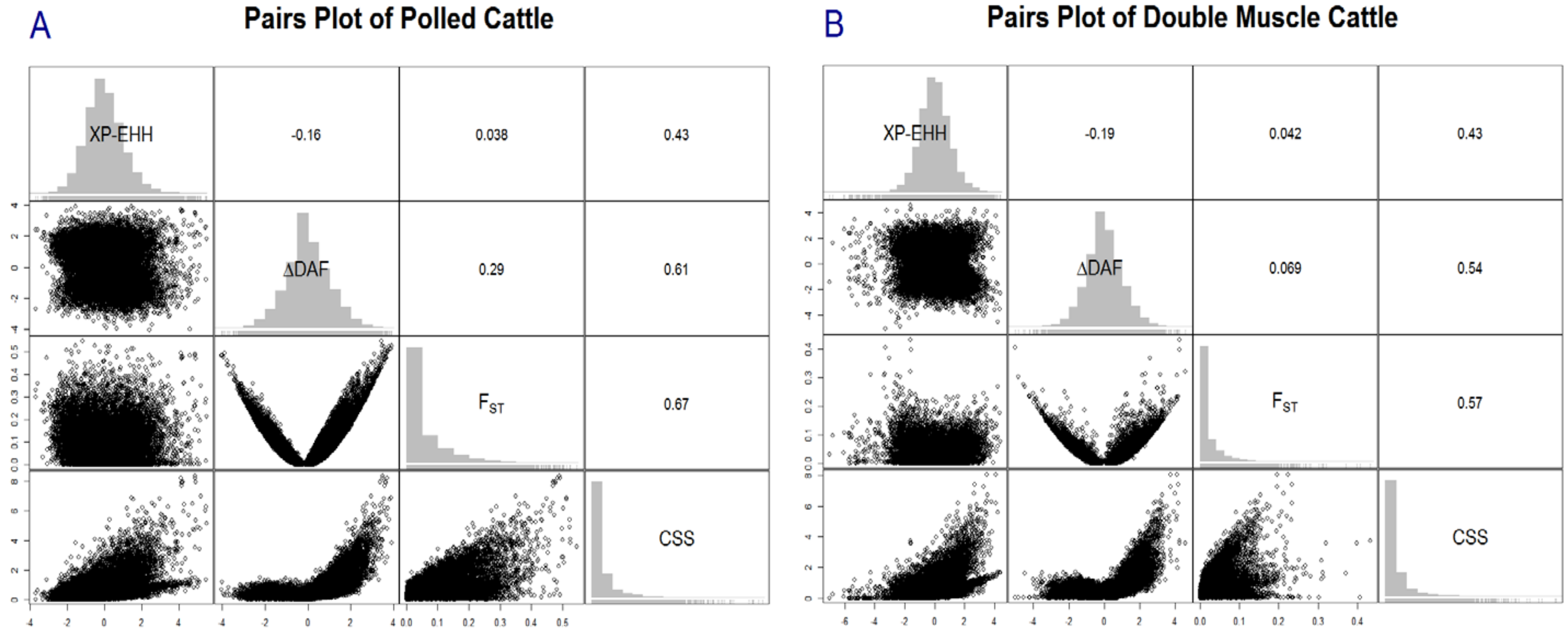

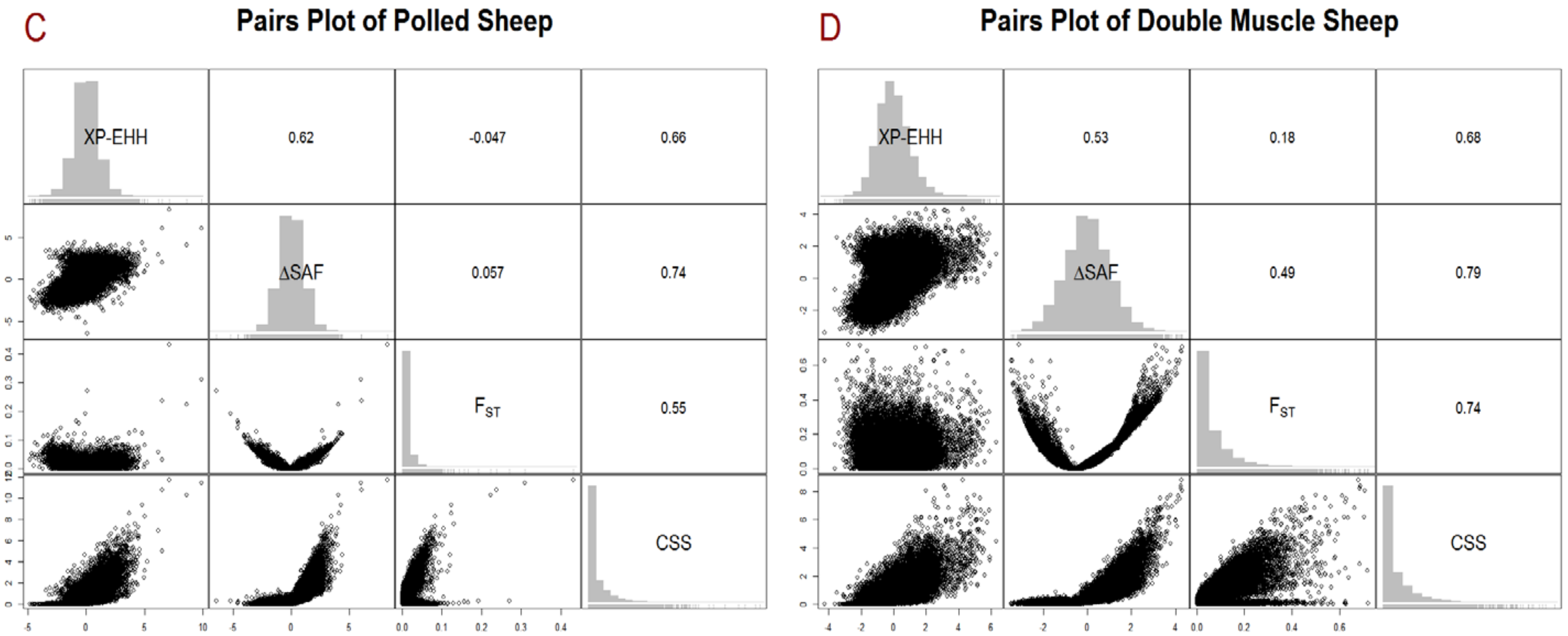

Figure S6. Genome-wide pairs plots (lower diagonals), histograms (diagonals) and correlations (upper diagonals) for constituent (XP-EHH, ΔSAF, F_{ST}) and composite selection signals (CSS) for polled (A), double muscle (B), cattle polledness (C) and double muscling (D) in sheep.
