# Supplementary material for: Composite selection signals can localize the trait specific genomic regions in multi-breed populations of cattle and sheep
Source: BMC Genet. 2014 Mar 17;15:34. doi: 10.1186/1471-2156-15-34 (PMC4101850; doi:10.1186/1471-2156-15-34)
Supplement: Additional file 10: Table S4 — Complete list of genomic regions and genes harbouring significant SNPs identified by four tests in four cohorts of cattle and sheep data. Cluster of minimum three significant SNPs within a window spanning 1 Mb genomic locations centred on a core SNP above the threshold (top 0.1%) in multiple tests (smoothed statistics) are reported and are compared with each other. [file 1471-2156-15-34-S10.pdf]

Table S4. Complete list of genomic regions and genes harbouring significant SNPs identified by four tests in four cohorts of cattle and sheep data. Cluster of minimum three significant SNPs within a window spanning 1 Mb genomic locations centred on a core SNP above the threshold (top 0.1%) in multiple tests (smoothed statistics) are reported and are compared with each other.

Region [a]	Chr	Position [b] (Mb)	Total SNPs [c] within the region	Number of Significant SNPs in clusters (Boundaries of the cluster in Mb)				Genes within the genomic regions [d], known genes [e] are bold	
				CSS	*XPEHH*	F_{ST}	*ΔDAF*	N	Genes
**A1**	1	1.01-2.63	14	**10*** (**1.51-2.13**)	**9** (**1.51-1.90**)	**1** (**1.63**)	-	15	CRYZL1, DONSON, SON, GART, TMEM50B, IFNAR1, IL10RB, IFNAR2, **OLIG1, C1H21orf62, GCFC1, SYNJ1,** C1H21orf59, C1H21orf63, URB1
A2	1	150.40-151.50	4	-	-	**4** (**150.90-151.00**)	-	4	PIGP, TTC3, DSCR3, DYRK1A
A3	4	64.05-65.22	3	-	-	-	**3** (**64.55-64.72**)	7	BMPER, MIR1814C, BBS9, NT5C3, FKBP9, KBTBD2, LSM5
A4	4	69.45-71.59	22	-	-	**22** (**69.95-71.09**)	-	10	SKAP2, SNX10, CBX3, HNRNPA2B1, NFE2L3, MIR148A, NPVF, CYCS, OSBPL3, DFNA5
A5	13	63.90-65.97	23	**18** (**64.40-65.47**)	**23*** (**64.66-65.86**)	**1** (**64.40**)	**5** (**63.91-64.40**)	26	RALY, EIF2S2, **ASIP,** AHCY, ITCH, DYNLRB1, MAP1LC3A, GGT7, ACSS2, GSS, MIR499, TRPC4AP, EDEM2, PROCR, EIF6, **UQCC, GDF5,** ERGIC3, SPAG4, CPNE1, RBM12, NFS1, ROMO1, RBM39, PHF20, SCAND1
A6	14	17.91-19.10	5	-	-	-	**5** (**18.41-18.60**)	8	FBXO32, WDYHV1, ATAD2, ZHX1, C14H8orf76, WDR67, DERL1, ZHX2
A7	14	23.78-25.61	11	**11** (**24.28-25.11**)	**7** (**24.79-25.35**)	**5*** (**24.52-24.79**)	**10*** (**24.28-24.79**)	12	SOX17, RP1, TMEM68, TGS1, LYN, RPS20, **PLAG1**, **CHCHD7,** SDR16C5, SDR16C6, PENK, IMPAD1
A8	19	10.40-12.23	4	-	-	**1** (**11.44**)	**4** (**10.90-11.73**)	12	GDPD1, YPEL2, DHX40, CLTC, PTRH2, VMP1, MIR21, TUBD1, RPS6KB1, RNFT1, TBX4, TBX2
A9	25	42.27-43.35	3	-	-	**3** (**42.77-42.85**)	-	9	GPR146, MIR2389, MIR339, COX19, ADAP1, GET4, GET4, PRKAR1B, PDGFA
**B1**	2	6.15-7.82	11	**10*** (**6.65-7.32**)	**11*** (**6.18-7.32**)	**3** (**6.70-6.79**)	-	9	**MSTN,** PMS1, ORMDL1, OSGEPL1, ASNSD1, SLC40A1, WDR75, MIR2917, COL3A1
B2	6	66.55-68.11	11	**11** (**67.05-67.61**)	**8** (**67.11-67.42**)	-	-	6	GABRA2, **COX7B2**, GABRA4, LOC536190, GABRB1, ATP10D, **FRYL**[f]

Additional file 10

Region [a]	Chr	Position [b] (Mb)	Total SNPs [c] within the region	Number of Significant SNPs in clusters (Boundaries of the cluster in Mb)				Genes within the genomic regions [d], known genes [e] are bold	
				CSS	*XPEHH*	F_{ST}	*ΔDAF*	N	Genes
B3	10	64.61-65.91	5	-	-	-	**5 (65.11-65.41)**	12	SQRDL, PLDN, C10H15orf48, MIR147, SPATA5L1, GATM, MIR2291, DUOX1, DUOXA1, SORD, FERMT2, DDHD1
B4	11	48.00-49.30	3	-	-	-	**3 (48.50-48.80)**	22	RMND5A, RNF103, CHMP3, KDM3A, REEP1, MRPL35, IMMT, PTCD3, POLR1A, ST3GAL5, ATOH8, GNLY, LOC616323, SFTPB, USP39, C11H2orf68, TMEM150A, RNF181, VAMP5, VAMP8, GGCX, MAT2A
B5	14	26.19-28.60	25	-	-	**25* (26.69-28.10)**	-	7	UBXN2B, CYP7A1, SDCBP, TOX, CA8, RAB2A, CLVS1
B6	16	44.49-46.05	11	**11 (44.99-45.55)**	**11 (44.99-45.55)**	**1 (45.55)**	-	12	**NMNAT1**, CTNNBIP1, PIK3CD, TMEM201, **SLC25A33**, SPSB1, MIR34A, **SLC2A5**, CA6, ENO1, **SLC45A1**, LOC786597
B7	17	49.08-50.24	3	-	-	-	**3 (49.58-49.74)**	2	GLT1D1, SLC15A4
B8	18	13.34-15.03	12	**5 (13.84-14.53)**	**3 (14.36-14.53)**	**1 (13.86)**	-	33	SLC7A5, CA5A, BANP, CYBA, MVD, SNAI3, RNF166, CTU2, MIR2327, APRT, GALNS, TRAPPC2L, ACSF3, CDH15, SLC22A31, SPG7, RPL13, CPNE7, DPEP1, CHMP1A, CDK10, SPATA2L, FANCA, SPIRE2, SPIRE2, TCF25, **MC1R,** TUBB3, DEF8, DBNDD1, GAS8, SHCBP1, VPS35
B9	20	30.65-31.80	3	-	-	-	**3 (31.15-31.30)**	12	FGF10, NNT, PAIP1, C20H5orf34, C20H5orf28, CCL28, HMGCS1, TRH, NIM1, ZNF131, ZNF131, SEPP1
B10	21	46.23-47.38	3	-	-	-	**3 (46.73-46.88)**		BRMS1L, MBIP, NKX2-1, NKX2-8, PAX9, SLC25A21
B11	22	46.16-47.40	6	-	**6 (46.66-46.90)**	-	-	1	CACNA2D3
B12	26	19.11-20.51	7	-	-	-	**7 (19.61-20.01)**		LOXL4, LOXL4, PYROXD2, MIR1287, HPS1, CNNM1, GOT1, NKX2-3, SLC25A28
C1	1	202.19-203.86	3	-	-	-	**3 (202.69-203.36)**	14	ITGB5, HEG1, SLC12A8, ZNF148, SNX4, OSBPL11, IQCG, LRCH3, FYTTD1, KIAA0226, MUC4, TNK2, TFRC, ZDHHC19

Region [a]	Chr	Position [b] (Mb)	Total SNPs [c] within the region	Number of Significant SNPs in clusters (Boundaries of the cluster in Mb)				Genes within the genomic regions [d], known genes [e] are bold	
				CSS	***XPEHH***	F_{ST}	***ΔDAF***	**N**	**Genes**
C2	3	172.57-174.00	6	-	-	-	**6 (173.07-173.50)**	49	XRCC6BP1, LOC100134274, CTDSP2, LOC645534, LOC645561, AVIL, TSFM, FAM119B, METTL1, CYP27B1, LOC100289240, LOC100289817, LOC100292982, CDK4, TSPAN31, AGAP2, LOC100130776, OS9, SVIP, B4GALNT1, LOC100293005, LOC100289202, LOC100289780, SLC26A10, GEFT, DTX3, PIP4K2C, KIF5A, DCTN2, MBD6, DDIT3, MARS, ARHGAP9, GLI1, INHBE, INHBC, R3HDM2, NXPH4, LRP1, STAT6, NAB2, TMEM194A, ZBTB39, GPR182, HSD17B6, PRIM1, NACA, NACA2, NACAP1, PTGES3
C3	4	50.52-51.85	6	-	-	-	**6 (51.02-51.35)**	16	FLJ36031, PIK3CG, PRKAR2B, HBP1, COG5, GPR22, DUS4L, BCAP29, LOC286002, SLC26A4, LOC100128737, CBLL1, ZNF645, SLC26A3, DLD, LAMB1
C4	10	24.84-27.59	5	-	-	**5 (25.34-27.09)**	-	18	FAM48B1, FAM48B2, LOC100294444, EXOSC8, ALG5, SMAD9, RFXAP, C13orf36, CCNA1, SPG20, SOHLH2, LSM3, LOC647302, DCLK1, NBEA, MAB21L1, BCL8, C2orf27A
**C5**	10	28.54-30.05	34	**26* (29.04-30.00)**	**17* (29.04-29.55)**	**34* (29.04-30.27)**	**5* (29.14-29.74)**	**9**	PDS5B, MDH1, N4BP2L2, N4BP2L1, ZAR1L, FRY, EEF1DP3, **RXFP2,** B3GALTL
C6	10	38.86-40.90	21	-	**21 (39.36-40.40)**	-	-	2	LMLN, PCDH9
C7	12	5.41-6.88	6	-	-	-	**6 (5.91-6.38)**	1	KCNT2
C8	13	66.97-68.50	9	**7 (67.47-68.00)**	-	**7 (67.47-68.00)**	**3 (67.86-67.95)**	17	C20orf186, C20orf71, PLUNC, CDK5RAP1, SNTA1, CBFA2T2, NECAB3, E2F1, PXMP4, ZNF341, CHMP4B, RALY, EIF2S2. **ASIP**, AHCY, TOMM20, LOC100129272, ITCH
C9	16	56.98-58.13	5	-	-	-	**5 (57.48-57.63)**	0	-

Additional file 10

Region [a]	Chr	Position [b] (Mb)	Total SNPs [c] within the region	Number of Significant SNPs in clusters (Boundaries of the cluster in Mb)				Genes within the genomic regions [d], known genes [e] are bold	
				CSS	*XPEHH*	F_{ST}	*ΔDAF*	N	Genes
C10	25	6.67-8.29	15	**14 (7.17-7.79)**	**10 (7.17-7.65)**	-	**2 (7.17-7.20)**	16	SLC35F3, C1orf31, TARBP1, IRF2BP2, LOC100290173, LOC100131463, SNORA14A, SNORA14B, RBM34, ARID4B, GGPS1, TBCE, B3GALNT2, LYST, NID1, **LRP4**[f]
D1	2	118.48-120.10	15	-	**15 (118.98-119.60)**	-	-	13	CLCN3, LOC100288206, LOC100290958, LOC100291623, NEK1, CBR4, PALLD, FLJ45445, LOC100132062, LOC100132287, ANXA10, HIATL1, LOC100130353
D2	2	119.62-122.30	23	**20 (120.12-121.80)**	**11* (121.06-121.80)**	**10 (120.12-120.90)**	**16 (120.12-121.80)**	26	ANXA10, HIATL1, LOC100130353, LGSN, OCA2, HERC2, LOC100290629, LOC390561, HERC2P2, HERC2P4, LOC728278, LOC646139, LOC100132101, LOC100289574, LOC100293165, LOC100294446, NIPA1, NIPA2, POLR2K, CYFIP1, TUBGCP5, IMP4, LOC644037, LOC645691, LOC100291878, PLEKHB2
D3	2	122.89-124.60	17	-	**17 123.39-124.10**	-	-	15	HS6ST1, LOC100293267, LOC100289081, UGGT1, SAP130, LOC439994, LOC642361, LOC100288164, LOC100293494, LOC642361, LOC100288907, LOC100291465, LOC728190, AMMECR1L, POLR2D
**D4**	2	124.25-128.05	54	**28* (124.75-127.55)**	**5 (125.97-126.31)**	**27 (124.69-127.58)**	**27 (124.69-127.61)**	47	UGGT1, SAP130, LOC439994, LOC642361, LOC100288164, LOC100293494, LOC642361, LOC100288907, LOC100291465, LOC728190, AMMECR1L, POLR2D, WDR33, LOC100288089, LOC100293529, LIMS2, GPR17, LIMS2, MYO7B, IWS1, PROC, MAP3K2, ERCC3, CYP27C1, BIN1, LOC100294448, NAB1, MFSD6, HIBCH, LOC100288000, LOC100290880, LOC100292514, C2orf88, **MSTN,** PMS1, ORMDL1, OSGEPL1, ANKAR, ASNSD1, SLC40A1, WDR75, COL5A2, COL3A1, PLGLA, PLGLB1, PLGLB2, GULP1
D5	5	63.23-64.63	11	-	-	**11 (63.73-64.13)**	**4 (63.73-64.13)**	12	ADRB2, ABLIM3, AFAP1L1, PCYOX1L, IL17B, CSNK1A1, CSNK1A1L, CSNK1A1P, FLJ41603, PPARGC1B, PDE6A, SLC26A2

[a] Prefix (A, B, C and D) with each region number represents the data set as defined in Table 1 (main text). Extracted list of 12 genomic regions, their positions, number of significant SNPs (for each test) and number of genes within each genomic region is shown in Table 2.

[b] Position of genomic regions includes a 0.5 Mb extension on both sides of boundaries of main cluster identified by CSS to compare constituent tests and count of genes. Large sized (> 1 Mb) regions are constituted by joining successive (<1 Mb apart) clusters.

[c] Total number of SNPs located within the boundaries of the largest cluster identified by any of 4 tests and their position exclude 0.5 Mb additions for gene investigation (shown in [b]). Total number of SNPs located within the boundaries of the main cluster identified by CSS only is shown in Table 3 of main text.

[d] Number (N) and list of genes mapped on bovine (UMD3.1) and ovine (OAR.v1) assemblies within the boundaries of genomic regions.

[e] Candidate genes with known functional/structural effects for a particular trait present in the contrasting panels of multiple breeds.

[f] Downstream genomic location (outside) of the listed gene from the region in Mb.

* Indicates the cluster of highest ranked SNPs (raw scores) for particular selection test.
