# Supplementary material for: Composite selection signals can localize the trait specific genomic regions in multi-breed populations of cattle and sheep
Source: BMC Genet. 2014 Mar 17;15:34. doi: 10.1186/1471-2156-15-34 (PMC4101850; doi:10.1186/1471-2156-15-34)
Supplement: Additional file 11: Figure S7 — Histograms of Mean Z, raw p-value and calibrated p-values distributions of the CSS: Histograms (top to bottom in each column) for polled cattle (column 1, red), double muscle cattle (column 2, green), polled sheep (column 3, purple) and double muscle sheep (column 4, blue). [file 1471-2156-15-34-S11.pdf]

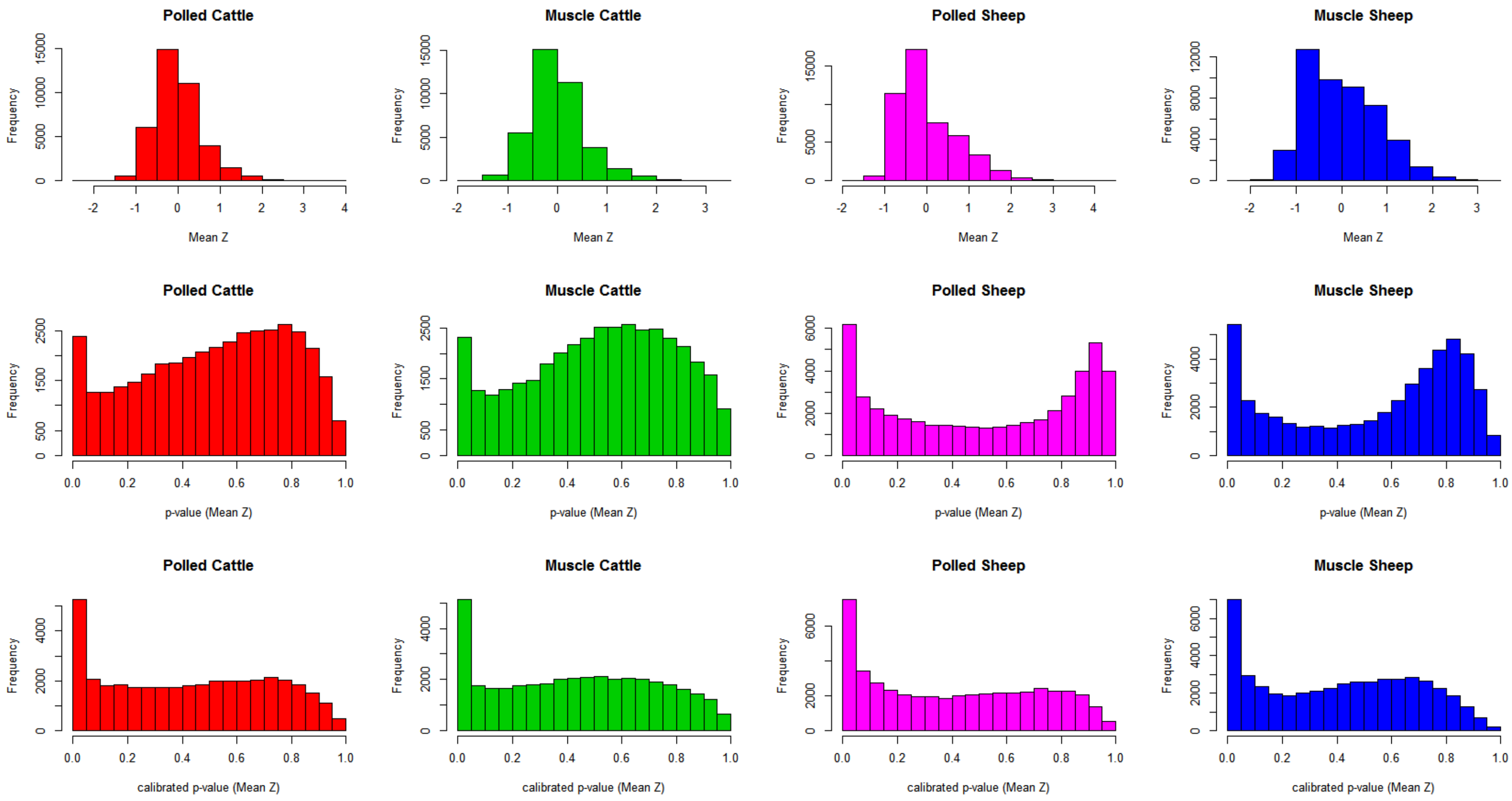


**Figure S7. Histograms of Mean *Z*, raw *p*-value and calibrated *p*-values distributions of the CSS:** Histograms (top to bottom in each column) for polled cattle (column 1, red), double muscle cattle (column 2, green), polled sheep (column 3, purple) and double muscle sheep (column 4, blue).
