# Supplementary material for: Composite selection signals can localize the trait specific genomic regions in multi-breed populations of cattle and sheep
Source: BMC Genet. 2014 Mar 17;15:34. doi: 10.1186/1471-2156-15-34 (PMC4101850; doi:10.1186/1471-2156-15-34)
Supplement: Additional file 12: Figure S8 — False discovery rate (FDR) against p-values: q-values were calculated from the calibrated p-values. Vertical dotted (……) and dashed (−−−−−) lines indicate calibrated p-values at 0.01 and 0.05, respectively. Horizontal dotted and dashed lines indicate q-values (FDR) at 0.05 and 0.1, respectively. [file 1471-2156-15-34-S12.pdf]

Additional file 12

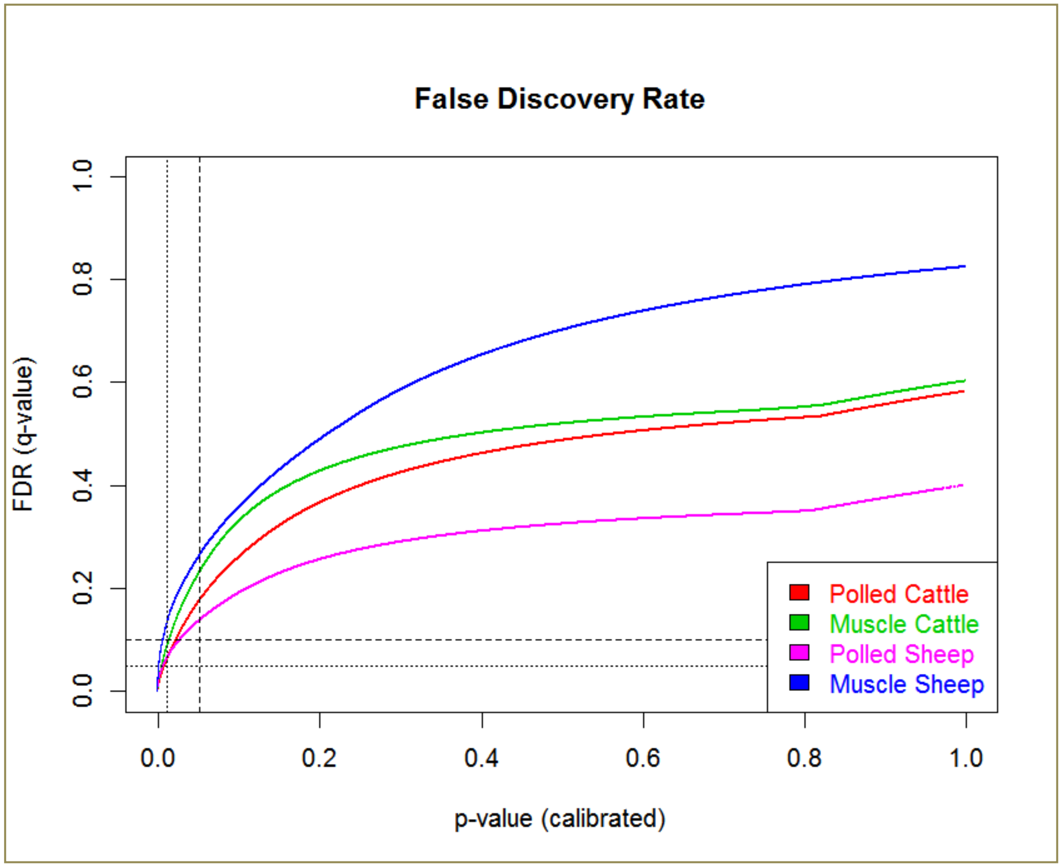

Figure S8. False discovery rate (FDR) against *p*-values: *q*-values were calculated from the calibrated *p*-values. Vertical dotted (……) and dashed (-----) lines indicate calibrated *p*-values at 0.01 and 0.05, respectively. Horizontal dotted and dashed lines indicate *q*-values (FDR) at 0.05 and 0.1, respectively.
