# Supplementary material for: Composite selection signals can localize the trait specific genomic regions in multi-breed populations of cattle and sheep
Source: BMC Genet. 2014 Mar 17;15:34. doi: 10.1186/1471-2156-15-34 (PMC4101850; doi:10.1186/1471-2156-15-34)
Supplement: Additional file 13: Table S5 — Selection signatures in European and African Bos taurus cattle populations. Complete list of selection signatures identified by composite (CSS) and constituent (XPEHH, FST, ΔDAF) selection tests. [file 1471-2156-15-34-S13.pdf]

Additional file 13

Table S5. Selection signatures in European and African *Bos taurus* cattle populations. Complete list of selection signatures identified by composite (CSS) and constituent (XPEHH, FST, ΔDAF) selection tests.

BTA	Location [a] (Mb)	Tests	Population	Genes [b]
**1**	1.76-2.13	CSS	European	SON, GART, LOC784171, TMEM50B, IFNGR2, IFNAR1, IL10RB, IFNAR2, LOC526226, **OLIG1, C1H21orf62, GCFC, SYNJ1,** C1H21ORF59, C1H21ORF63, URB1, MRAP, MIS18A
**1**	131.1-131.64	CSS, XPEHH, FST	African	COPB2, MRPS22, LOC616668, LOC616673, LOC783600, LOC616698, LOC616710, FOXL2, PIK3CB, LOC100138061, FAIM, LOC100301027, LOC616840, CEP70, ESYT3, MRAS, TXNDC6, ARMC8, DBR1, A4GNT, LOC100337454, DZIP1L
**1**	150.90-151.00	ΔDAF	African	CLDN14, LOC100337405, LOC100337422, HLCS, PIGP, TTC3, DSCR3, DYRK1A
**1**	157.95-158.2	CSS, ΔDAF	African	LOC100139888, LOC100297040, [No gene]
**1**	82.0-82.2	CSS, XPEHH	African	DGKG, ETV5, LOC616971, TRA2B, IGF2BP2, SENP2, LIPH, TMEM41A, MAP3K13, EHHADH, LOC100140254, LOC615631
**3**	10.52-10.71	ΔDAF	African	DUSP23, CRP, APCS, LOC508986, LOC616952, LOC509261, LOC530601, LOC504688, FCER1A, LOC538552, MPTX, DARC, CADM3, IFI16, LOC519294, LOC526177, LOC517209, LOC514540, LOC505209, SPTA1
**4**	1.3-2.0	CSS	African	No gene
**4**	51.97-52.01	ΔDAF	European	ST7, LOC100337386, CAPZA2, MET, LOC781951, CAV1, CAV2, LOC100296613, TES
**5**	26.60-26.91	FST	African / European	HOXC6, LOC100295249, LOC100295214, MIR196A-2, HOXC11, HOXC12, HOXC13, CALCOCO1, ATP5G2, ATF7, NPFF, TARBP2, MAP3K12, PCBP2, PRR13, AMHR2, SP1, SP7, AAAS, C12orf10, PFDN5, ESPL1, MFSD5, RARG, ITGB7, ZNF740, LOC516241, SOAT2, IGFBP6, SPRYD3, TENC1, EIF4B, KRT18, KRT8, KRT78, KRT79, KRT4, LOC100299984, LOC100336907, KERIA, LOC100336698, LOC100301161
**6**	53.1-53.4	CSS, ΔDAF	African	No gene
**6**	88.8-89.3	CSS	African	SLC4A4, GC, ADAMTS3, COX18
**7**	39.5-40.0	CSS, XPEHH	African	COMMD10, ARL10, MIR1271, NOP16, HIGD2A, CLTB, FAF2, RNF44, CDHR2, GPRIN1, SNCB, EIF4E1B, TSPAN17, UNC5A, HK3, UIMC1, LACTB, LOC533921, ZNF346, FGFR4, LOC100336707, NSD1, RAB24, PRELID1, MXD3, LMAN2, RGS14, SLC34A1, PFN3, F12, GRK6, PRR7, DBN1, PDLIM7, DOK3, DDX41, FAM193B, LOC509184, LOC100139419
**7**	43.39-43.42	XPEHH	European	LOC787932, LOC787946, LOC787963, LOC788011, LOC788055, LOC616716, LOC788079, LOC506034, LOC509217, LOC514645, LOC539568, LOC510877, LOC788190, LOC511141, LOC616938, LOC515313, LOC615374, LOC510603, LOC788287, LOC618395, LOC510631, LOC788323, LOC507971, LOC509922, LOC788361, LOC524160, LOC517722, LOC518474, LOC100299275, MGC137030, MGC148293
**8**	43.80-44.40	CSS, ΔDAF	African	DMRT2, DMRT3, DMRT1, LOC100336820, KANK1, DOCK8, LOC513962, LOC100300568, FOXD4L1, PGM5
**8**	83.05-83.57	FST	African / European	LOC531757, MIR2475, MIR23B, MIR27B, MIR24-1, FANCC, LOC785608, TSPY, LOC100336570, FANCC, PTCH1, LOC789977

Additional file 13

BTA	Location [a] (Mb)	Tests	Population	Genes [b]
**9**	43.86-44.04	FST	African / European	PDSS2, BEND3, C9H6ORF203, MGC127538, QRSL1, RTN4IP1, LOC526200, ATG5, LOC100336714, PRDM1
**10**	30.27-30.65	ΔDAF	European	FMN1, GREM1, SCG5, ARHGAP11A, GJD2, ACTC1, AQR, LOC786537, ATPBD4
**11**	98.93-99.31	FST	African / European	SH2D3C, CDK9, FPGS, ENG, LOC100337088, AK1, ST6GALNAC6, ST6GALNAC2, PIP5KL1, DPM2, FAM102A, NAIF1, SLC25A25, PTGES2, LCN2, C11H9orf16, CIZ1, DNM1, MIR199B, GOLGA2, LOC616324, TRUB2, COQ4, SLC27A4, URM1, CERCAM, ODF2, GLE1, SPTAN1, WDR34, SET, PKN3, LOC505354, ZER1, TBC1D13, ENDOG, LOC100138783, CCBL1, LRRC8A, PHYHD1, DOLK, NUP188, SH3GLB2, FAM73B, DOLPP1, CRAT, PPP2R4, IER5L
**13**	11.58-12.15	CSS, XPEHH	European	LOC100337090, LOC100298513, LOC526041, LOC505099, LOC100337293, LOC509875, **CAMK1D**, **CDC123**, NUDT5, SEC61A2, DHTKD1, UPF2, LOC781700, ECHDC3
**14**	1.46-1.80	CSS, XPEHH, FST, ΔDAF	European	LOC788476, LOC100336014, LOC100295750, LOC100299291, LOC100294758, ZNF250, C14H8orf33, ZNF34, RPL8, ZNF7, COMMD5, LOC532363, MIR2308, LOC618766, LRRC24, LRRC14, RECQL4, MFSD3, GPT, PPP1R16A, LOC100294916, FOXH1, LOC789523, CYHR1, NFKBIL2, SLC39A4, CPSF1, ADCK5, GPR172B, FBXL6, LOC789629, SCRT1, **DGAT1**, HSF1, BOP1, SCXB, HEATR7A, LOC523023, LOC509113, LOC509114, MAF1, SHARPIN, CYC1, GPAA1, EXOSC4, OPLAH, LOC100141215, LOC618305, GRINA, PARP10, LOC786966, MIR2309, LOC100337278, NRBP2, PUF60, LOC100295316, LOC506831, LOC524974, MAPK15, LOC100138046, ZNF623, LOC100295394, TSTA3
**14**	17.75-18.46	ΔDAF	European	TMEM65, FER1L6, LOC507013, ANXA13, KLHL38, FBXO32, WDYHV1, ATAD2, ZHX1, LOC539014, LOC521399, WDR67, DERL1, ZHX2
**14**	24.15-24.79	CSS, FST, ΔDAF	European	XKR4, TMEM68, TGS1, LYN, RPS20, MOS, **PLAG1, CHCHD7**, SDR16C5, SDR16C6, PENK
**14**	79.36-80.08	ΔDAF	African	ATP6V0D2, MIR2311, LOC100196897, CA2, CA3, CA1, CA1, CA13, MGC133577, E2F5, LRRCC1, SLC7A13, LOC506670, RALYL
**15**	77.50-77.68	XPEHH	African	CREB3L1, DGKZ, MDK, CHRM4, AMBRA1, HARBI1, ATG13, ARHGAP1, ZNF408, LOC100336386, LOC781101, F2, CKAP5, MIR2319A, MIR2319B, LRP4, LOC617614, LOC509025, C15H11orf49
**16**	43.37-45.55	CSS, XPEHH, FST	European	MAD2L2, FBXO6, FBXO44, FBXO2, PTCHD2, UBIAD1, MTOR, **ANGPTL7**, EXOSC10, SRM, MASP2, TARDBP, CASZ1, PEX14, DFFA, CORT, APITD1, PGD, LOC100337161, UBE4B, RBP7, **NMNAT1**, LOC100337372, CTNNBIP1, CLSTN1, **PIK3CD**, TMEM201, **SLC25A33**, **SPSB1**, H6PD, MIR34A, GPR157, **SLC2A5**, CA6, **ENO1**, **RERE**, LOC100295969, **SLC45A1**, LOC786597
**19**	10.20-11.44	XPEHH	African	Sep-04, LOC616486, LOC100297075, TEX14, MGC137055, RAD51C, TDGF1, PPM1E, TRIM37, FAM33A, MIR454, MIR301A, PRR11, MGC143117, GDPD1, YPEL2, AK3L1, DHX40, CLTC, PTRH2, TMEM49, MIR21, TUBD1, RPS6KB1, RNFT1, MED13, INTS2, LOC787304, BRIP1, TBX4
**19**	10.90-10.92	ΔDAF	European	GDPD1, YPEL2, AK3L1, DHX40, CLTC, PTRH2, TMEM49, MIR21, TUBD1, RPS6KB1, RNFT1, MED13, INTS2
**20**	22.67-22.70	ΔDAF	African	MIER3, LOC540253, MAP3K1, [No gene], ANKRD55
**21**	13.69-13.74	ΔDAF	African	NDUFB4, LOC100337442, [No gene]
**22**	49.70-50.01	ΔDAF	African	WDR82P1, PPM1M, TWF2, TLR9, ALAS1, WDR51A, DUSP7, RPL29, ACY1, ABHD14A, ABHD14B, PCBP4, GPR62, LOC507426, RRP9, IQCF1, IQCF5, IQCF2, LOC783987, LOC100125949, LOC785460, LOC618550, GRM2, TEX264, LOC784245, VPRBP, RBM15B, MANF, DOCK3, MAPKAPK3, CISH, HEMK1, C22H3ORF18, CACNA2D2
**29**	35.96-36.09	ΔDAF	European	NTM, [No gene], TMEM45B

[a] location represents the boundaries of the significant SNP clusters.

Additional file 13

[b] Genes in red colour are located within the significant cluster and genes in black colour are included at a 0.5 Mb flanking regions on both sides of the significant cluster. Gene highlighted as bold have known functional role for various phenotypes under selection.
