# Supplementary material for: Composite selection signals can localize the trait specific genomic regions in multi-breed populations of cattle and sheep
Source: BMC Genet. 2014 Mar 17;15:34. doi: 10.1186/1471-2156-15-34 (PMC4101850; doi:10.1186/1471-2156-15-34)
Supplement: Additional file 14: Figure S9 — Genome-wide comparison of using SNP genotype data from all breeds (Total: 46 breeds and N = 847), breeds with minimum 10 samples (Total: 26 breeds and N = 753) and breeds with minimum 20 samples (Total: 20 breeds and N = 652) for computing CSS (A), XP-EHH (B), ΔDAF (C) and FST (D) for European Bos taurus cattle. [file 1471-2156-15-34-S14.pdf]

Additional file 14

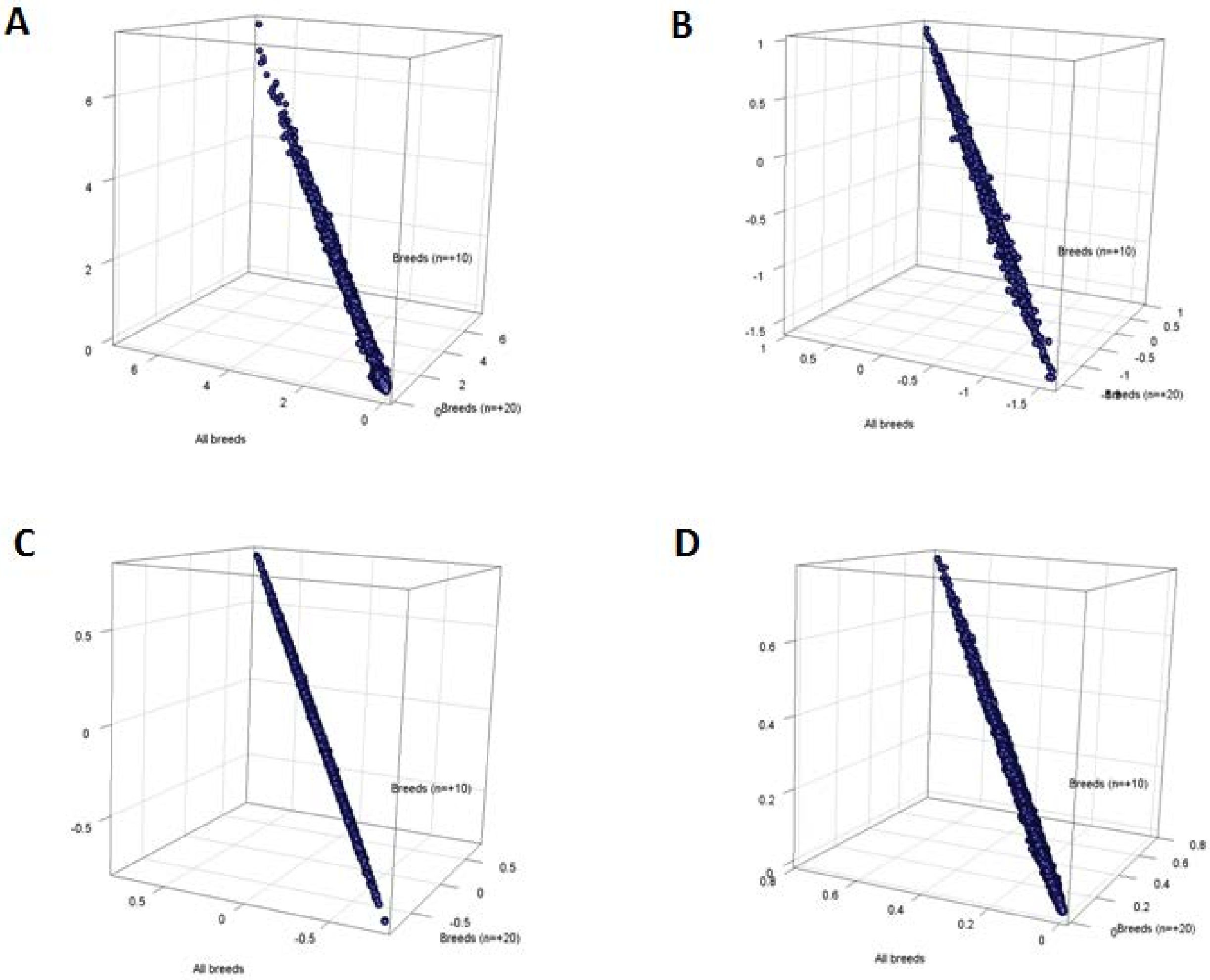

Figure S9. Genome-wide comparison of using SNP genotype data from all breeds (Total: 46 breeds and N= 847), breeds with minimum 10 samples (Total: 26 breeds and N=753) and breeds with minimum 20 samples (Total: 20 breeds and N=652) for computing CSS (A), XPEHH (B), ΔDAF (C) and F_{ST} (D) for European *Bos taurus* cattle.
