# Supplementary material for: Composite selection signals can localize the trait specific genomic regions in multi-breed populations of cattle and sheep
Source: BMC Genet. 2014 Mar 17;15:34. doi: 10.1186/1471-2156-15-34 (PMC4101850; doi:10.1186/1471-2156-15-34)
Supplement: Additional file 15: Figure S10 — Chromosome-wise comparison of average derived allele frequencies (A) and average minor allele frequencies (B) between European and African Bos taurus cattle breeds. [file 1471-2156-15-34-S15.pdf]

Additional file 15

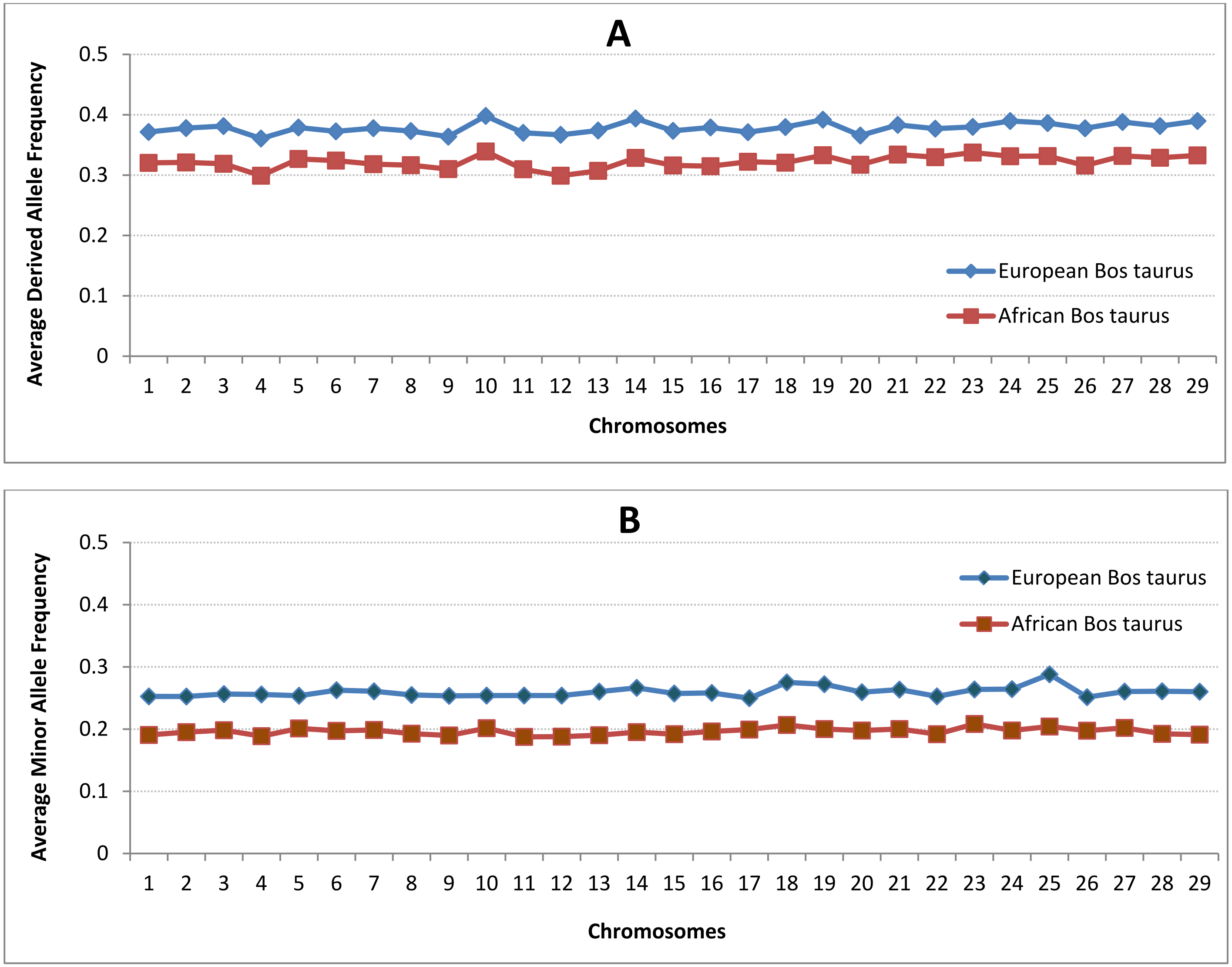

Figure S10. Chromosome-wise comparison of (A) average derived allele frequencies (DAF) and (B) average minor allele frequencies (MAF) between European and African *Bos taurus* cattle breeds. In addition to the ΔDAF computation for cattle analyses, we also compared the chromosomal average DAF and MAF between geographic groups of European and African cattle to understand the genome-wide selection pressures.
